# Hardware-aware knowledge-distilled CNN for real-time classification of plaque-associated angiographic findings on FPGA

**DOI:** 10.3389/fdgth.2026.1847917

**Published:** 2026-09-03

**Authors:** Nazarkar Pravalika, Jabeena Afthab, Vetriveeran Rajamani

**Affiliations:** School of Electronics Engineering (SENSE), Vellore Institute of Technology, Vellore, Tamil Nadu, India

**Keywords:** coronary angiography, FPGA-based acceleration for deep learning, hardware-aware CNN, knowledge distillation, plaque-associated angiographic findings, Vitis HLS

## Abstract

Coronary artery disease (CAD) is one of the leading causes of global mortality, necessitating accurate assessment of coronary artery stenosis and **plaque-associated angiographic findings** from coronary angiography images. Although deep learning-based diagnostic models have demonstrated high predictive capability, their deployment on resource-constrained embedded platforms remains challenging because of computational complexity and memory requirements. To address these limitations, this study proposes a knowledge-distillation-based hardware-aware framework that transfers discriminative knowledge from a high-capacity teacher network to a lightweight convolutional neural network (CNN). The proposed framework integrates software-level localization of **plaque-associated angiographic findings** with FPGA-oriented optimization. Using a 90% training and 10% testing split, the proposed model achieved an accuracy of **98.64%**, precision of **99.06%**, recall of **97.82%**, PPV of **0.99**, NPV of **0.98**, MCC of **0.96**, and an AUC of **0.9940**. And under 10-fold cross-validation, the model achieved a mean accuracy of **97.53%** **±** **0.27%**, precision of **97.82%**, recall of **96.94%**, PPV of **0.98**, NPV of **0.97**, MCC of **0.95**, and a mean AUC of **0.9897**, which confirms that our proposed model exhibits high robust performance. For hardware realization, the optimized student CNN was deployed on a **Xilinx Zynq UltraScale** **+** **MPSoC FPGA** using the **Vitis High-Level Synthesis (HLS)** toolchain. The implemented accelerator achieved a **kernel-level inference latency of 10.6 µs** and a **Peak On-Chip Kernel Throughput of approximately 94,339 inferences/s**, while maintaining balanced hardware resource utilization and low power consumption. Overall, the proposed framework demonstrates accurate classification of **plaque-associated angiographic findings** with efficient FPGA deployment.

## 1 Introduction

Coronary artery disease (CAD) is one of the major causes of mortality worldwide (1, 2), characterized by the progressive accumulation of atherosclerotic plaques in the coronary arteries, which narrow the arteries and reduce perfusion of the heart muscle (3). Early detection of plaque-induced stenosis is important for timely diagnosis and effective treatment planning. Coronary angiography is commonly used to diagnose anomalies of the coronary vessels. It is also used to identify stenosis associated with atherosclerotic plaque. Manual analysis of images obtained via angiography is time-consuming and highly dependent on clinicians’ expertise, leading to diagnostic inconsistencies. These factors have led to the development of automated systems for classifying plaque-associated angiographic findings and diagnosing coronary artery disease using computer-aided techniques. Recent advances in deep learning, specifically CNNs, have greatly improved image analysis and classification in medical image processing applications (4–6). CNNs have been successfully applied to the diagnosis of coronary artery stenosis, plaque characterisation, and CAD diagnosis across different imaging modalities (5, 7, 8). Moreover, CNNs have been successfully used to classify plaque-associated angiographic findings (9). These CNN models have shown high accuracy in classifying coronary plaque-associated angiographic findings; however, most of the models use large networks with millions of parameters, which are computationally intensive. These requirements are unfavorable for the use of CNNs in real-time coronary classification of plaque-associated angiographic findings in resource-constrained environments. Model compression techniques, specifically pruning, quantisation, and knowledge distillation, have been explored to reduce network complexity while maintaining high accuracy in classifying plaque-associated angiographic findings. Knowledge distillation is an effective technique for model compression, in which knowledge from a large network is transferred to a compact one. In knowledge distillation, the compact network is trained on the soft outputs of the large network, enabling effective feature representation with significantly fewer parameters.

Previous studies have demonstrated that knowledge distillation can reduce computational cost while preserving classification accuracy, making it suitable for resource-constrained applications (10). It also enables the development of compact deep learning models with reduced parameter counts, making them suitable for Field-programmable gate array (FPGA) based acceleration in resource-constrained systems (10–12). In parallel with algorithmic optimisation, hardware acceleration platforms have gained more attention for efficient deep learning prediction. FPGAs provide deterministic latency, high parallelism, and improved energy efficiency compared with conventional CPU and GPU architectures (13, 14). FPGA-based accelerators enable customised computational pipelines (15) and optimised memory access patterns, which can significantly improve real-time deep learning prediction performance for medical image analysis in classifying plaque-associated angiographic findings. Several studies have investigated FPGA architectures for accelerating convolutional neural networks using pipelined and parallel processing techniques (11, 12, 16, 17). Furthermore, hardware-aware optimisation processes such as quantisation and high-level synthesis have been employed to improve resource efficiency in FPGA implementation (18, 19).

Despite these advances, most existing FPGA-based implementations primarily focus on conventional CNN architectures and do not incorporate knowledge distillation or compression strategies. Conversely, many knowledge distillation studies evaluate models only in software environments without considering hardware deployment constraints such as latency, memory bandwidth, and FPGA resource utilization. Consequently, integrated frameworks that combine knowledge-distilled neural networks with hardware-efficient FPGA implementations for coronary artery classification of plaque-associated angiographic findings remain limited. Unlike existing FPGA-based compressed CNN implementations developed for generic image classification tasks (11, 12, 15, 20, 21), the proposed framework specifically targets coronary artery classification of plaque-associated angiographic findings through a segmentation-aware teacher–student knowledge distillation strategy integrated with FPGA-oriented optimisation (26). The developed framework combines vascular feature-guided teacher learning, lightweight student CNN optimisation, parameter pruning, 8-bit fixed-point quantisation, and FPGA-based inference acceleration on the Zynq UltraScale + platform to achieve low-latency, resource-efficient classification while preserving diagnostic performance.

To address these limitations, this study presents a two-stage knowledge-distilled deep learning framework. In the first stage, plaque regions are identified from coronary angiography images at the software level to isolate diagnostically relevant regions. In the second stage, a compact student CNN obtained through knowledge distillation from a high-capacity teacher network performs classification of plaque-associated angiographic findings. The distilled student model is synthesised using the Vitis High-Level Synthesis (HLS) design flow and deployed on a Xilinx Zynq UltraScale + FPGA platform to achieve low-latency, resource-efficient inference for real-time classification of plaque-associated angiographic findings.

Coronary angiography remains the clinical reference standard for evaluating luminal narrowing (22) caused by atherosclerotic disease. While it does not directly visualise plaque morphology within the vessel wall, angiographic features such as stenotic lesions and lumen irregularities provide important indicators of underlying atherosclerotic burden assessment and CAD risk evaluation. Therefore, automated analysis of expert-annotated angiographic images can assist in identifying plaque-associated angiographic findings, thereby supporting the CAD assessment.

The Key contributions of this work are outlined as follows:

- A Knowledge distillation-based framework for classification of plaque-associated angiographic findings that reduces the complexity of the teacher CNN by transferring knowledge to a lightweight student CNN architecture, while maintaining diagnostic accuracy for coronary artery classification.
- A hardware-aware student CNN architecture, derived from the distilled model, optimised for FPGA deployment using a fully pipelined Vitis HLS design.
- A real-time FPGA implementation enabling low-latency inference for the classification of plaque-associated angiographic findings from coronary angiography images, achieving high-throughput performance with efficient FPGA resource utilisation for real-time edge-level medical deployment.
- Comprehensive experimental validation across multiple evaluation protocols, including accuracy, precision, recall, and Mathews Correlation Coefficient (MCC), demonstrating robustness under class- imbalanced conditions.

The remainder of this paper is organised as follows: Section 2 presents the proposed knowledge distillation- based framework for classification of plaque-associated angiographic findings and FPGA deployment using Vitis HLS design flow. Section 3 covers the software and hardware results of the proposed model. Finally, Section 4 presents the discussion, along with the potential directions for future work, and Section 5 presents the conclusion.

## 2 Proposed methodology

The proposed framework consists of two stages: the training stage and the hardware deployment stage. In the training stage, a high-capacity teacher convolutional neural network (CNN) is trained using the ARCADE Phase 1 Task 2 (Stenosis Detection) dataset. During preprocessing, the official COCO (Common Objects in Context) polygon annotations are converted into segmentation masks for teacher model training, while the corresponding expert annotations provide supervisory labels for the distilled student CNN to learn discriminative representations associated with plaque-associated angiographic findings. GAN-based data augmentation is applied only to the training partition to improve data diversity and reduce overfitting. After training, the distilled student CNN is deployed on the FPGA platform using the Vitis HLS design flow for hardware implementation of the classification of plaque-associated angiographic findings. The hardware implementation flow has been specifically designed to ensure the correctness of the design and the efficient mapping of the proposed model onto the FPGA architecture. Initially, the C-level simulation has been carried out to validate the functional correctness of the proposed model. Subsequently, the high-level synthesis has been applied to the proposed model to transform the algorithm into the Register Transfer Level (RTL) representation. This has been done to allow the application of optimisations such as pipelining and parallel execution to the model to improve the efficiency of the computational model. Finally, the RTL co-simulation has been carried out to verify the consistency of the proposed design with the high-level model. The design has then been exported in the form of synthesizable RTL (Verilog) code for the implementation of the proposed model on the FPGA platform. The proposed model has been integrated in the form of the RTL representation obtained from the Vitis-HLS flow. Subsequently, using the Vivado tool, the proposed design has been implemented on the Xilinx Zynq UltraScale + FPGA platform.

**Figure 1:**
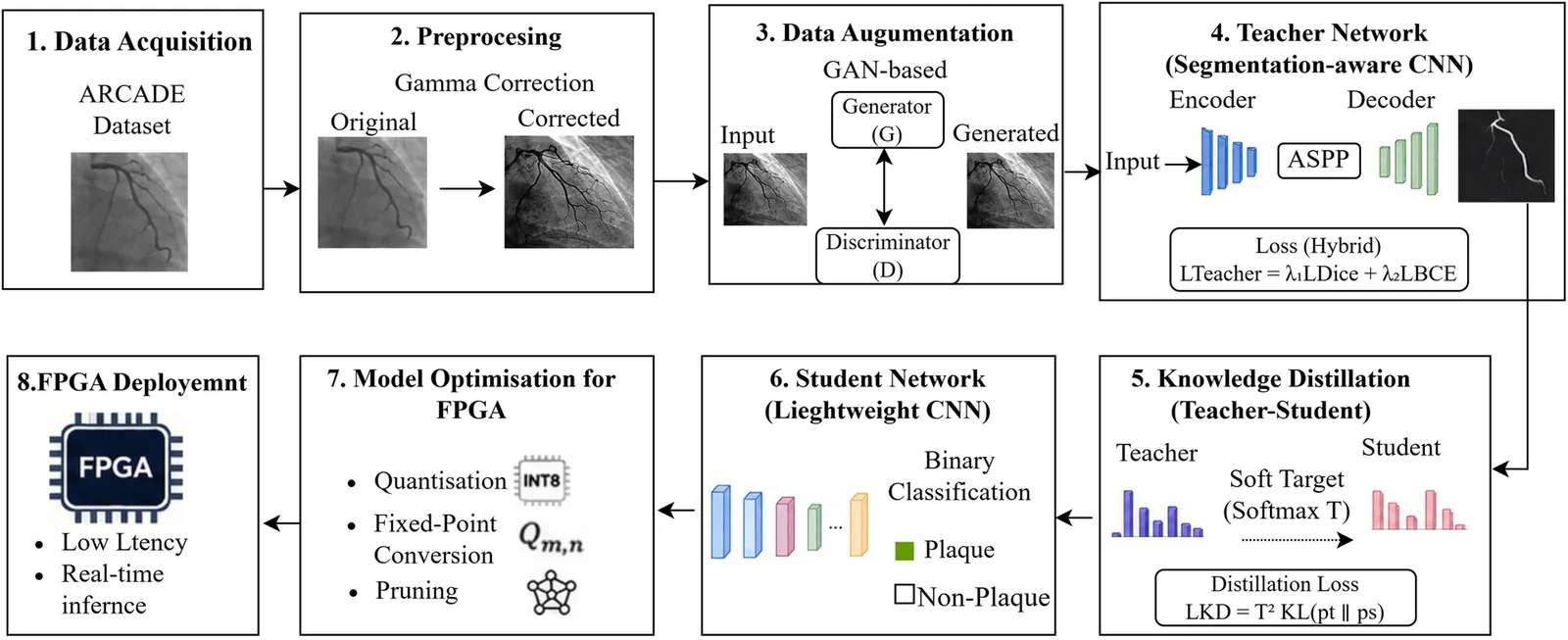
Illustrates the comprehensive framework of the proposed knowledge-distillation-based FPGA deployment pipeline for real-time classification of plaque-associated angiographic findings.

The complete hardware design flow is implemented to ensure the efficient hardware realisation of the proposed model with minimal latency and to ensure the reliable real-time classification of plaque-associated angiographic findings. The complete flow of the proposed framework has been presented in Figure 1.

Figure 1 shows the complete offline training and online FPGA inference workflow of the proposed framework. Initially, coronary X-ray angiography images from the publicly available ARCADE Phase 1 Task 2 (Stenosis Detection) dataset (512 × 512 resolution) undergo gamma correction to enhance vessel contrast. The corresponding polygon annotations and category labels provided in the Common Objects in Context (COCO) annotation files are directly used to generate the ground-truth labels for teacher model training and student model supervision without any additional manual annotation or relabeling. Subsequently, GAN-based augmentation is applied exclusively to the training data to improve data diversity and reduce overfitting, increasing the effective training dataset to approximately 9,600 images. The augmented images are then provided to the segmentation-aware teacher CNN with an encoder-decoder architecture and Atrous Spatial Pyramid Pooling (ASPP)-based multi-scale feature extraction. The teacher network is trained using hybrid Dice and BCE loss functions to learn rich semantic representations, which are subsequently transferred to the lightweight student CNN through knowledge distillation using KL-divergence optimisation. The distilled student CNN performs computationally efficient binary classification of plaque-associated angiographic findings using reduced convolutional blocks and Global Average Pooling. Finally, the optimised student model is deployed on the FPGA using the Vitis HLS design flow, incorporating signed INT8 quantisation, fixed-point conversion, and hardware optimisation to enable low-latency real-time classification. The subsequent sections describe each stage of the proposed framework in detail.

### 2.1 Dataset description

The proposed framework was evaluated using the publicly available ARCADE Phase-1 Task-2 (Stenosis Detection) dataset (23), which consists of **1,500 coronary X-ray angiography images**, including **1,000 training images, 200 public validation images, and 300 private test images** with corresponding expert-provided annotations in COCO format. The publicly available training and validation partitions were used for model development, while the official private test partition was reserved for benchmark evaluation. To improve the diversity of the training data and reduce overfitting, **GAN-based augmentation was applied exclusively to the selected training partition of the ARCADE Phase 1 Task 2 dataset**, increasing the effective training dataset to approximately **9,600 images**, while the validation dataset consisted exclusively of the original ARCADE Phase 1 Task 2 images. Since the publicly available ARCADE Phase 1 Task 2 dataset does not provide **patient-level or sequence-level identifiers**, patient-wise or sequence-wise data partitioning could not be performed. Therefore, **image-level data partitioning** was adopted throughout this study. To obtain a robust and reliable performance evaluation, **10-fold cross-validation** was employed, where approximately **90% of the images were used for training and the remaining 10% were used for validation in each fold**, ensuring that each image was used exactly once for validation during the cross-validation process.

#### 2.1.1 Image-level label generation

The binary image-level classification labels were derived directly from the official expert-provided **ARCADE Phase 1 Task 2** annotations without any additional manual labeling, region-of-interest extraction, or modification of the original annotations. Images containing expert-annotated stenotic regions were assigned to the **plaque-associated angiographic findings** class (positive class), whereas images without annotated stenotic regions were assigned to the **non-plaque-associated angiographic findings** class (negative class). Thus, the binary image-level labels were generated exclusively from the official dataset annotations without introducing any external datasets or additional annotations.

The original **ARCADE Phase 1 Task 2** dataset exhibited class imbalance. The original training partition contained **1,280 positive** and **2,560 negative** samples, while the validation and test partitions contained **256 positive and 512 negative** samples, and **386 positive and 772 negative** samples, respectively. Overall, the original unaugmented dataset comprised **1,922 positive** and **3,844 negative** samples.

To mitigate the influence of class imbalance during model training, **GAN-based data augmentation was applied exclusively to the positive training samples**, increasing the number of positive training samples from **1,280 to 9,600**, while the **2,560 negative training samples remained unchanged**. The validation and test partitions were retained in their original form without augmentation. Consequently, all validation and testing results reported in this study were obtained exclusively from the original ARCADE Phase 1 Task 2 validation and test datasets, thereby ensuring an unbiased evaluation of the proposed framework.

### 2.2 GAN-based data augmentation

Medical imaging datasets are often limited in size, which can increase the risk of overfitting during deep learning training. To improve the diversity of the training data, **Generative Adversarial Networks (GANs)** (4, 5) were employed to generate synthetic coronary X-ray angiography images. A GAN consists of two competing neural networks: a **generator (G)**, which produces synthetic images from random noise, and a **discriminator (D)**, which distinguishes real images from generated images. Through this adversarial learning process, the generator progressively produces more realistic samples while the discriminator improves its ability to differentiate real and synthetic images. The minimax optimization objective of the GAN is defined in Equation 1.

$$\underset{G}{\min}\underset{D}{\max}V(D,G)=E_{x∼p_{data}(x)}[\log D(x)]+E_{z∼p_{z}(z)}[\log(1-D(G(z)))]$$ (1)

where,

- G – Generator network producing synthetic coronary angiography images
- D – Discriminator network distinguishing real and generated images
- X – Real coronary angiography image sampled from the dataset
- Z – Random noise vector sampled from a latent distribution
- G(z) – Synthetic image generated from noise
- D(x) – Probability that image x is classified as real
- D(G(z)) – Probability that the generated image is classified as real

To improve the diversity of the training data and mitigate class imbalance, **GAN-based augmentation was applied exclusively to the positive training samples** of the **ARCADE Phase 1 Task 2** dataset, increasing the number of positive training samples from **1,280 to 9,600**, while the **2,560 negative training samples remained unchanged**. For each fold of the 10-fold cross-validation, the original dataset was first partitioned into training and validation sets, after which GAN-generated synthetic images were created only from the corresponding positive training samples. The generated synthetic images were used solely during model training and were **not included in the validation or test datasets**. Consequently, all reported confusion matrices and performance metrics were obtained exclusively from the original unaugmented validation and test datasets, thereby minimizing the possibility of data leakage and ensuring an unbiased evaluation of the proposed framework. Experimental results demonstrated that GAN-based augmentation improved the classification performance of the proposed knowledge-distilled student CNN. Furthermore, the ablation study showed that excluding GAN-generated samples reduced the classification performance, confirming the effectiveness of GAN-based augmentation in improving model generalization, as presented in Table 3.

#### 2.2.1 Evaluation protocol

Since the publicly available **ARCADE Phase 1 Task 2** dataset does not provide patient-level or sequence-level identifiers, patient-wise or sequence-wise data partitioning could not be performed. Therefore, image-level partitioning was adopted throughout this study. To obtain a robust performance evaluation, the proposed framework was assessed using a **10-fold cross-validation** strategy, where approximately **90%** of the images were used for training and the remaining **10%** for validation in each fold, ensuring that every image was used exactly once for validation during the complete cross-validation process. Furthermore, **GAN-based augmentation was applied exclusively to the positive training samples within each fold**, while the validation and test datasets remained unaugmented to minimize the possibility of data leakage and ensure an unbiased evaluation.

### 2.3 Gamma correction

Coronary angiography images often exhibit low contrast and non-uniform illumination, which may obscure vessel boundaries and plaque-associated regions. To improve the visibility of vascular structures and enhance diagnostic features, gamma correction (4) is applied as an image-pre-processing step. Gamma correction performs a nonlinear intensity transformation that adjusts pixel intensity values while preserving the anatomical structure of the coronary vessels. The gamma correction transformation is expressed in Equation 2.

$$I_{out}=(I_{in}{)}^{\gamma }$$ (2)

where,

- I_in_ represents the input pixel intensity of the angiography image
- I_out_ represents the corrected output pixel intensity
- *γ* represents the gamma parameter controlling contrast adjustment

When 0 < *γ* < 1, darker regions in the image are enhanced, which improves the visibility of coronary vessels and plaque regions that typically appear in low-intensity areas. The improvement in vessel-to- background contrast after gamma correction can be expressed (5) in Equation 3.

$$C_{\gamma }=(I_{v}^{\gamma }-I_{b}^{\gamma })/I_{b}^{\gamma }$$ (3)

where,

- I*_γ_* represents vessel pixel intensity
- I*b* represents background pixel intensity
- C*_γ_* denotes the contrast ratio after gamma correction

The gamma parameter was selected empirically based on a preliminary visual assessment and diagnostic feature enhancement analysis of coronary angiography images from the ARCADE dataset. Gamma values ranging from 0.6 to 1.2 were evaluated to determine their effect on vessel visibility, plaque-associated region enhancement, and background contrast preservation. Experimental observations indicated that *γ*= 0.8 provided the best balance between vessel enhancement and noise suppression while preserving anatomical structures. Therefore, this value was selected as an optimal pre-processing parameter for subsequent knowledge distillation and FPGA-based classification stages. The enhanced angiographic images were then used as input for the proposed teacher-student knowledge distillation framework.

### 2.4 Teacher–student knowledge distillation framework

**Figure 2:**
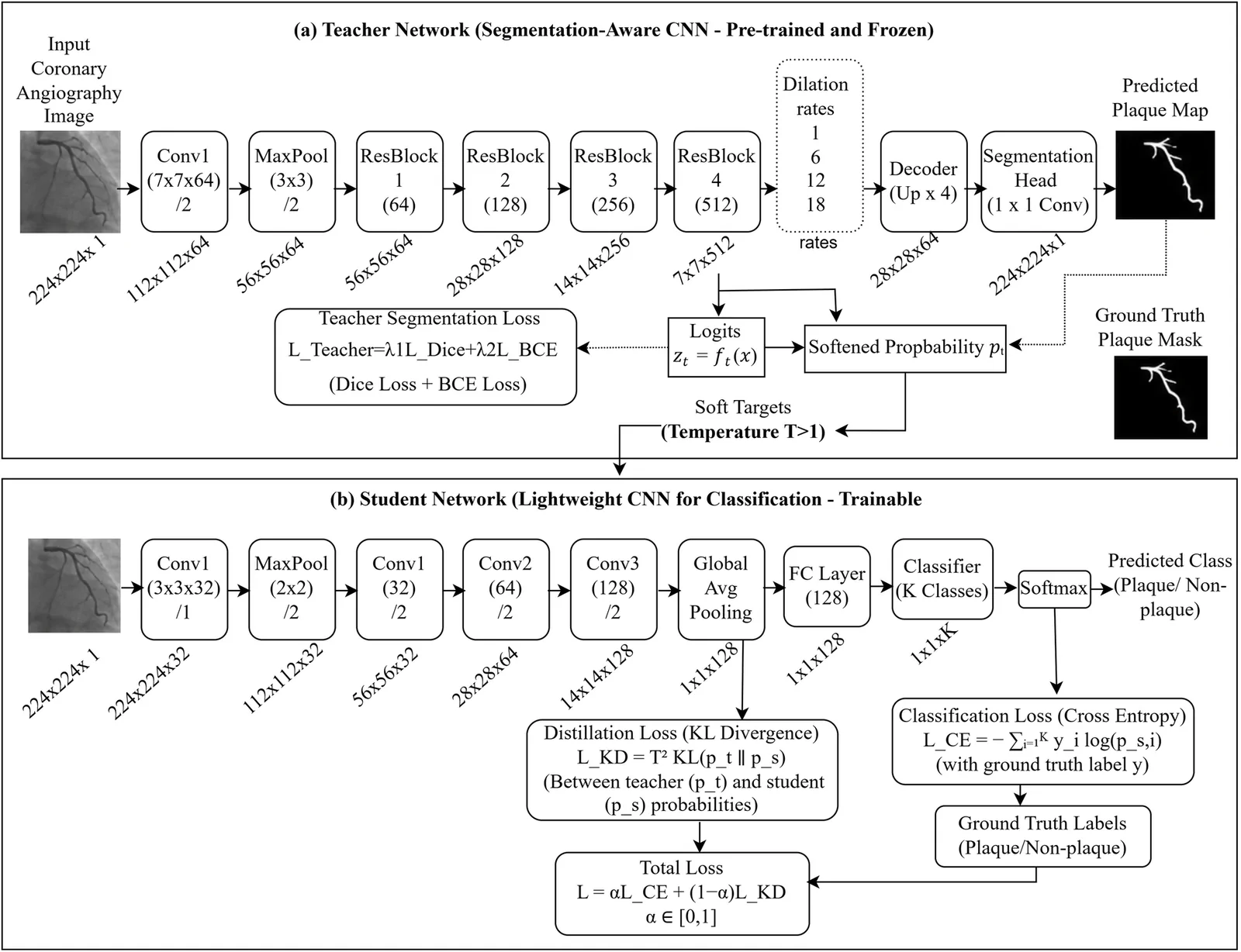
Segmentation-aware teacher–student knowledge distillation framework for lightweight FPGA-efficient classification of plaque-associated angiographic findings.

The proposed teacher–student knowledge distillation framework reduces model complexity while preserving diagnostic accuracy, as illustrated in Figure 2. The framework consists of a pre-trained teacher network with frozen weights and a lightweight student network optimised for resource-efficient FPGA deployment. During training, the same coronary angiography image of size (224 × 224 × 1) is simultaneously provided to both the teacher and student networks.

In the teacher branch, the input image first passes through a 7 × 7 convolution layer followed by a max-pooling operation for low-level feature extraction and spatial reduction. The extracted feature maps are subsequently processed through four residual blocks with feature dimensions of 64, 128, 256, and 512 channels, respectively, enabling hierarchical extraction of vascular and angiographic feature representations.

To improve multi-scale contextual feature learning, dilated convolution operations with dilation rates of 1, 6, 12, and 18 are incorporated to enlarge the receptive field while preserving spatial information. The encoded feature maps are then passed through a decoder with ×4 upsampling to restore spatial resolution, followed by segmentation using a 1 × 1 convolution-based segmentation head for expert-annotated stenotic region prediction. The predicted segmentation map is compared with the corresponding ground-truth segmentation mask using a hybrid segmentation loss composed of Dice loss and Binary Cross-Entropy (BCE) loss, defined by Equation 4.

$$L_{Teacher}=\lambda_{1}L_{Dice}+\lambda_{2}L_{BCE}$$ (4)

where,

- L_Teacher_ denotes the overall teacher training loss
- L_Dice_ represents the Dice loss function used for overlap-based segmentation of expert-annotated regions
- L_BCE_ represents the Binary Cross-Entropy loss for pixel- level classification of angiographic findings associated with atherosclerosis, and
- *λ*_1_ and *λ*_2_ are weighting coefficients controlling the contribution of each loss component

After completion of segmentation training, the predicted segmentation maps are spatially aggregated to obtain image-level class logits for each input image. These logits are subsequently converted into temperature-scaled soft probability distributions using the SoftMax function, which serve as the soft targets for supervising the student network during knowledge distillation. Consequently, the student network learns from the teacher's image-level probability distributions rather than directly from the pixel-wise segmentation masks, enabling effective knowledge transfer from the segmentation-based teacher network to the image-level binary classification student network. The softened probability distribution is defined by Equation 5.

$$P_{i}=\exp(z_{i}/T)/\underset{j}{\sum }\exp(z_{j}/T)$$ (5)

Where,

- P_i_ denotes the softened probability corresponding to class i
- Z_i_ represents the logit of the current class i
- *Σ*_j_ represents summation over all classes indexed by j.
- Z_j_ represents logits of all classes used in the denominator normalisation term,
- T denotes the temperature scaling parameter. In this study, the temperature parameter was empirically set to T=4 during training to generate softened probability distributions for knowledge transfer.
- exp (·) represents the exponential function used in the SoftMax computation.

The temperature parameter enables preservation of inter-class semantic relationships and provides informative supervisory signals for knowledge transfer. Simultaneously, the student branch employs a lightweight CNN architecture designed for low-complexity FPGA deployment. The student network consists of successive convolution layers with 32, 64, and 128 feature channels, followed by global average pooling, a fully connected layer, and a SoftMax classifier for binary classification of plaque-associated angiographic findings. During training, the student model learns from both ground-truth class labels and softened probability distributions generated by the teacher network. The student network is optimised using a composite knowledge distillation loss function is defined by Equation 6.

$$L_{KD}=\lambda_{1}L_{CE}(y,p_{s})+\lambda_{2}T^{2}KL(\;p_{t}∥p_{s})+\lambda_{3}|F_{t}-F_{s}{|}^{2}$$ (6)

where,

- L*_KD_* denotes the total knowledge distillation loss used for student optimisation
- L*_CE_* represents the Cross-Entropy loss between ground-truth labels (y) and student predictions (p*_s_*)
- KL(p*_t_* ∥ p*_s_*) denotes the Kullback–Leibler divergence between teacher and student soft probability distributions
- $|F_{t}-F_{s}{|}^{2}$ represents the feature alignment loss, enforcing similarity between intermediate feature representations of the teacher and student networks.
- *λ*_1_, *λ*_2_, and *λ*_3_ are weighting coefficients controlling the contribution of each optimisation term The weighting coefficients were empirically selected as $\lambda_{1}=\;0.5$, $\lambda_{2}=\;0.3$, and $\lambda_{3}=\;0.2$ to balance the contributions of the cross-entropy, KL divergence, and feature alignment losses during student optimisation.
- T denotes the temperature scaling parameter used for probability softening.

The feature alignment loss is computed between the final latent feature representations extracted immediately before the prediction layers of the teacher and student networks, enabling the student network to learn semantically consistent representations from the teacher. Although the teacher and student networks employ different architectures, they were designed to produce compatible latent feature representations with identical dimensionality at the distillation stage. Consequently, feature alignment was performed directly on the extracted latent representations without introducing additional feature projection or dimensionality transformation layers.

The student network was trained for 500 epochs with a batch size of 32 using the Adam optimizer and an initial learning rate of 0.001. During student optimisation, a dropout rate of 0.5 and an L2 regularization coefficient of 0.001 were employed to improve model generalization and reduce overfitting. These training hyperparameters were empirically selected to achieve stable convergence and improved classification performance during the knowledge distillation process.

**The tensor dimensions of the teacher and student networks are summarised as follows.** Both networks receive an input coronary angiography image of size **224** **×** **224** **×** **1**. In the teacher network, the feature maps progress through dimensions of **112** **×** **112** **×** **64**, **56** **×** **56** **×** **64**, **56** **×** **56** **×** **64**, **28** **×** **28** **×** **128**, **14** **×** **14** **×** **256**, and **7** **×** **7** **×** **512**, followed by the decoder **28** **×** **28** **×** **64**, and segmentation head **224** **×** **224** **×** **1** to generate the final segmentation map. In the lightweight student network, the corresponding feature maps are **224** **×** **224** **×** **32**, **112** **×** **112** **×** **32**, **56** **×** **56** **×** **32**, **28** **×** **28** **×** **64**, and **14** **×** **14** **×** **128**, followed by global average pooling to produce a **1** **×** **1** **×** **128** latent feature representation before the fully connected layer and SoftMax classifier.

The combined optimisation strategy enables the lightweight student CNN to learn discriminative representations associated with atherosclerotic angiographic findings from the high-capacity teacher network while maintaining low computational complexity. This facilitates efficient FPGA-based deployment with low-latency inference suitable for real-time classification of angiographic findings associated with coronary atherosclerosis.

### 2.5 Evaluation metrics

The performance of the proposed framework classification of plaque-associated angiographic findings is evaluated using standard metrics derived from the confusion matrix (8), including accuracy, positive predictive value (PPV), negative predictive value (NPV), sensitivity, specificity, and the Matthews correlation coefficient (MCC), as defined in Equations (7)–(11), respectively.

$$Accuracy=\frac{TP+TN}{TP+TN+FP+FN}$$ (7)

$$Precision=\frac{TP}{TP+FP}$$ (8)

$$Recall=\frac{TP}{TP+FN}$$ (9)

$$NPV=\frac{TN}{TN+FN}$$ (10)

$$MCC=\frac{TP\times TN-FP\times FN}{\sqrt{\left(TP+FP)(TP+FN)(TN+FP)(TN+FN\right)}}$$ (11)

where,

- T P – plaque-affected coronary arteries correctly classified as plaque-affected.
- T N – healthy coronary arteries correctly classified as healthy.
- FP – healthy coronary arteries incorrectly classified as plaque-affected.
- FN – plaque-affected coronary arteries incorrectly classified as healthy.

The above evaluation metrics assess the classification performance of the proposed framework for plaque-associated angiographic findings. Although the proposed framework demonstrates reliable classification performance in software, real-time deployment requires efficient hardware acceleration to achieve low latency, reduced computational complexity, and efficient FPGA resource utilisation. Therefore, the optimised distilled student model is translated into a hardware-compatible C++ code for Vitis HLS, and subsequently deployed on the target FPGA using the Vivado design suite.

The training procedure of the proposed knowledge distillation framework is summarised in the following Algorithm 1.

Algorithm 1 **Knowledge distillation training.**

1: Input: D={(x,y)}, Teacher (T), Student (S), temperature (T), weights (λ1,λ2,λ3), learning rate *η*, epochs E, batch size B.

2: **Output:** Trained student model (S*)              ▷ initialise weights

3: Initialise student parameters (*θ*s)

4: for e = 1 to E do

5: Shuffle (D) and split into mini-batches (B_k_)           ▷ random batching

6: for each batch (x, y) ∈ B_k_ do

7: Z_T_ ← T(x), $p_{T}=softmax(z_{T}/T)$                ▷ teacher output (frozen)

8: Z_S_ ← S(x), $p_{S}=softmax(z_{S}/T)$              ▷ student output

9: $L_{CE}\leftarrow \sum ylog(softmax(z_{S}))$               ▷ classification loss

10: $L_{KL}=\mathrm{KL}(\;p_{T}∥p_{S})$                  ▷ distillation loss

11: $\;\;\;L_{F}=|F_{T}-F_{S}{|}^{2}$                   ▷ feature alignment

12: $L=\lambda_{1}L_{CE}+\lambda_{2}T^{2}L_{KL}+\lambda_{3}L_{F}$               ▷ total loss

13: $\;\;\;\theta_{s}=\theta_{s}-\eta \nabla_{\theta_{s}}L$                    ▷ update student

14: end for

15: end for

16: return (S∗)

### 2.6 FPGA-based implementation of the distilled student model

**Figure 3:**
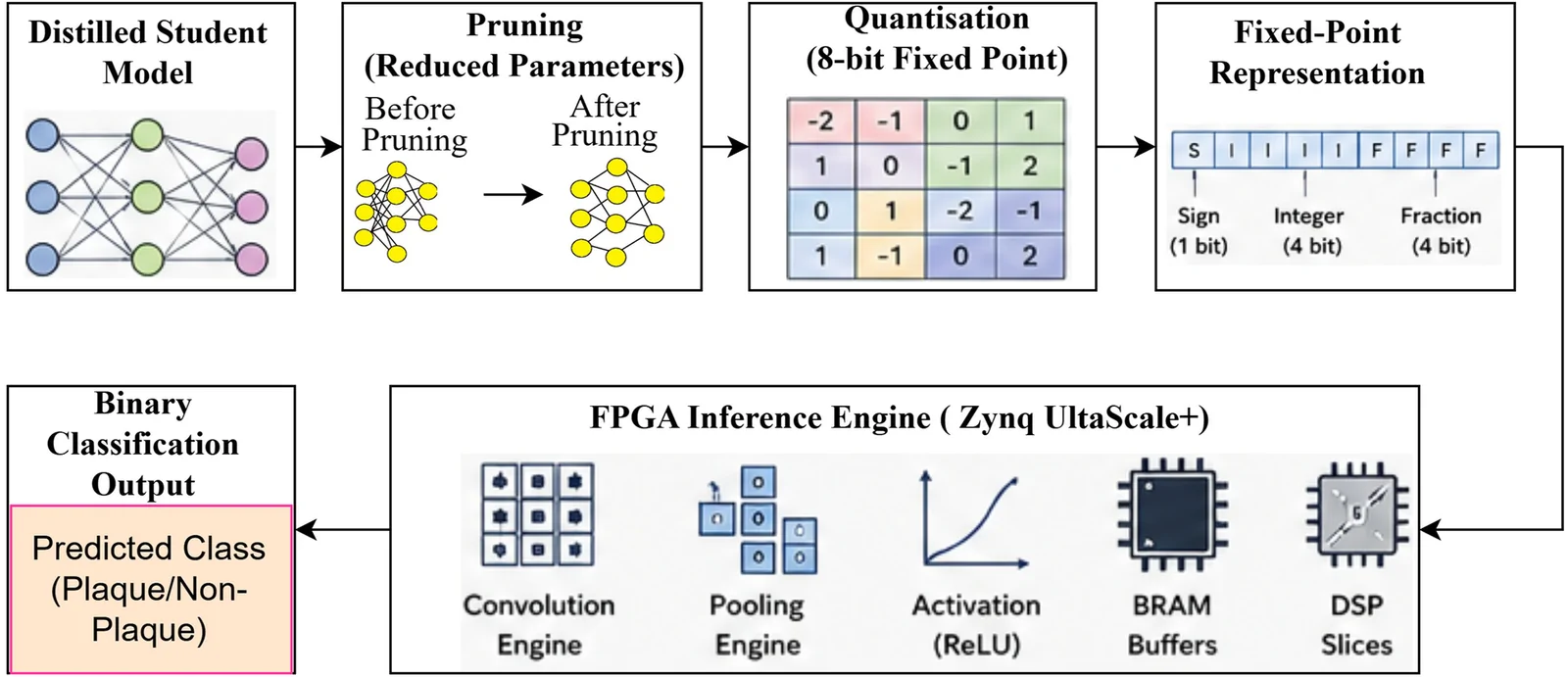
Hardware-software co-design architecture of the proposed knowledge-distilled FPGA accelerator for real-time coronary atherosclerotic classification of angiographic findings associated with atherosclerosis.

The FPGA-based deployment architecture of the proposed framework is illustrated in Figure 3. After completion of teacher–student knowledge distillation training, the distilled student CNN model is selected for FPGA deployment because of its computational complexity and efficient resource utilisation. As shown in Figure 3, the FPGA deployment pipeline begins with the distilled student CNN extracted from the complete teacher-student framework.

To improve hardware efficiency, the distilled student model was first optimized using magnitude-based weight pruning, in which weights with the smallest absolute magnitudes were removed. An overall pruning ratio of approximately 30% was employed, followed by fine-tuning using the same training configuration and knowledge distillation framework until validation convergence to recover any performance degradation caused by pruning. Subsequently, signed INT8 post-training quantization (PTQ) was applied prior to FPGA deployment. MinMax calibration was performed using a representative calibration dataset sampled from the validation partition to determine the scaling factors and zero-points required for integer quantization. These optimization procedures reduced the computational complexity and memory requirements of the student model while preserving its classification performance for efficient FPGA implementation.

The quantised parameters are represented using a fixed-point format consisting of sign, integer, and fractional bit, as illustrated in the fixed-point representation block. This representation enables efficient FPGA-compatible arithmetic using fixed-point computation instead of computationally expensive floating-point operations.

The processed fixed-point parameters are deployed to the FPGA inference engine implemented on the Zynq UltraScale + platform. The inference engine consists of dedicated convolution and pooling modules responsible for hierarchical feature extraction and spatial dimensionality reduction. The extracted features are subsequently processed through ReLU activation modules to introduce non-linearity during inference computation. In addition, BRAM buffers are used for intermediate feature-map storage and data reuse, while DSP slices perform high-speed multiply–accumulate operations (MAC) required during convolution processing.

Finally, the FPGA inference engine generates binary classification outputs corresponding to the plaque associated angiographic findings (positive) and non-plaque-associated angiographic findings (negative) prediction classes. The overall hardware pipeline shown in Figure 3 demonstrates the integration of distilled learning, parameter pruning, fixed-point quantisation, and FPGA-oriented inference acceleration for efficient real-time coronary artery classification of angiographic findings associated with atherosclerosis on the Zynq UltraScale + platform. The architecture achieves low latency, high throughput, and efficient DSP and BRAM utilisation for real-time CAD detection. To enable efficient hardware implementation, the high-level C++ model is synthesised into optimised RTL using Vitis HLS design flow, as detailed in the following section.

### 2.7 High-level synthesis using Vitis-HLS

**Figure 4:**
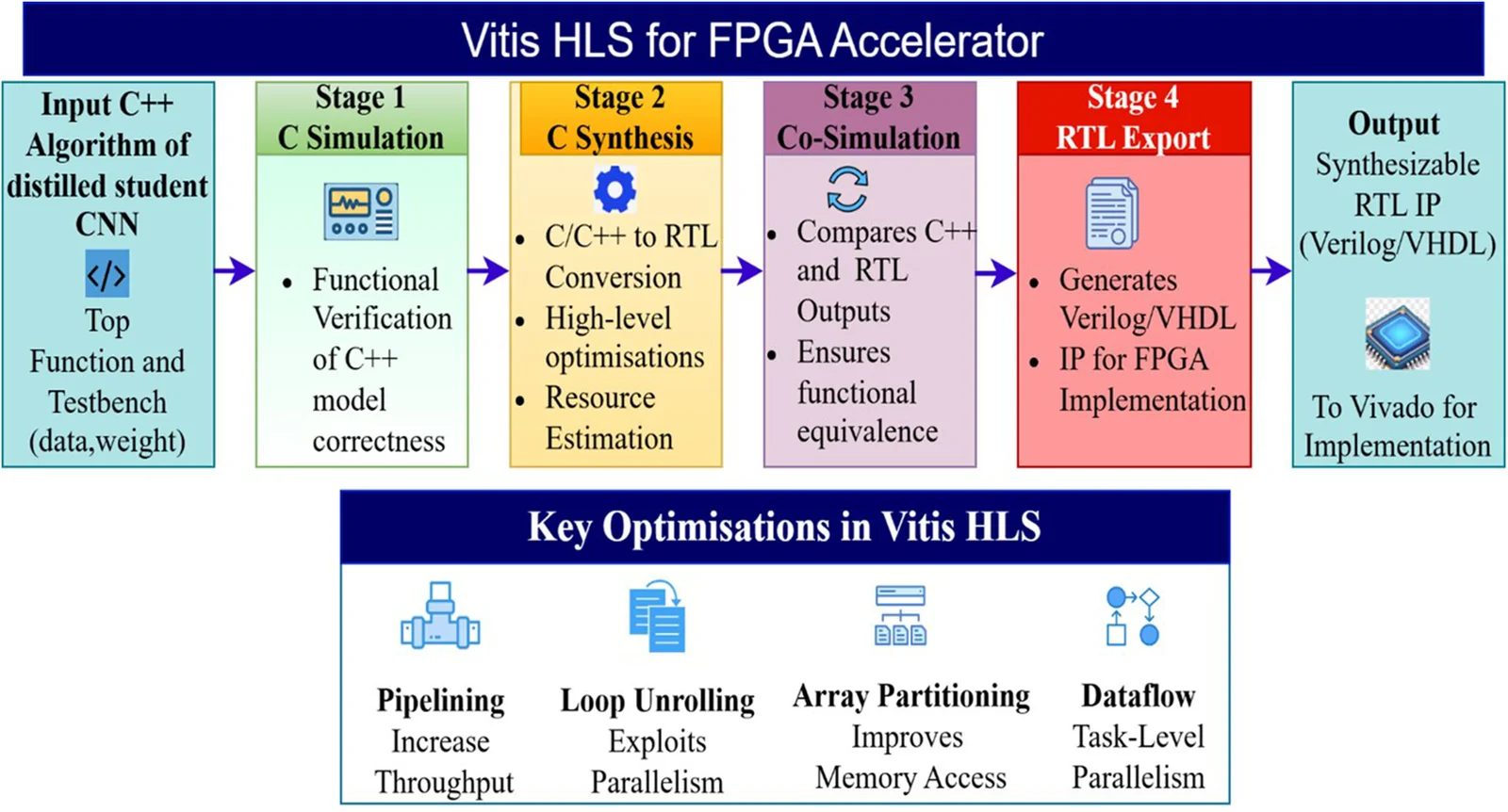
Vitis HLS design flow for generating an FPGA accelerator from high-level C++ implementation of the distilled student CNN.

To enable FPGA deployment, the distilled student CNN is implemented in C++ and synthesised using Vitis-HLS. The C++ implementation describes convolution, feature extraction, and classification operations required for classifying plaque-associated angiographic findings. The Vitis-HLS tool transforms the high-level C++ description of the distilled student model into Register Transfer Level (RTL) hardware modules for FPGA deployment. During synthesis, computational loops and data dependencies are optimised to enable pipelined and parallel execution, thereby reducing latency and improving inference efficiency for classifying plaque-associated angiographic findings (14, 19). HLS-based implementations are widely adopted for deep learning accelerators because they support efficient hardware implementation of neural network operations while preserving design flexibility (24). Figure 4 illustrates the Vitis HLS design flow adopted in this work, showing the transformation of the high-level C++ model into synthesizable RTL for FPGA implementation.

The Vitis HLS design flow begins with the high-level C++ implementation of the distilled student CNN, comprising convolution, ReLU, pooling, and fully connected operations. In the first stage, C Simulation is performed to verify the functional behaviour of the model using representative input data. In the second stage, C Synthesis, the high-level C++ description is converted into RTL hardware modules, where optimisation techniques, including loop unrolling and dataflow pipelining, are applied to improve parallelism and reduce latency. In the third stage, Co-Simulation, is performed to verify the functional equivalence between the generated RTL design and the original C++ implementation. In the final stage, RTL Export, the synthesizable Verilog RTL is generated and exported to the Vivado Design Suite for FPGA implementation. The overall HLS flow enables efficient hardware implementation of the distilled student CNN, achieving high throughput and low latency for real-time classification of plaque-associated angiographic findings. The performance of the synthesised FPGA accelerator is evaluated using latency and throughput metrics.

Latency denotes the time required for the FPGA accelerator to classify a single coronary angiography image into positive or negative classes and is expressed in Equation 12.

$$Latency=\frac{C_{img}}{\;f_{clk}}$$ (12)

Throughput denotes the number of coronary angiography images processed per second by the distilled student CNN on the FPGA, and is expressed in Equation 13.

$$Throughput=\frac{\;f_{clk}}{C_{img}}$$ (13)

where,

- $C_{img}$ is the number of clock cycles required to process one coronary angiography image using the distilled student CNN inference pipeline.
- $f_{clk}$ denotes the operating clock frequency of the FPGA accelerator.

The RTL design generated by Vitis-HLS is subsequently exported for FPGA implementation and hardware validation.

### 2.8 Vivado-based implementation and hardware execution

After RTL generation using Vitis HLS design flow, the distilled student CNN accelerator is imported into the Vivado Design Suite for FPGA implementation. The generated RTL modules are packaged as a reusable IP core and integrated into a system-level hardware design (25). Design constraints, including clock frequency and pin assignments, are specified using the Xilinx Design Constraints (XDC) file. Vivado then generates a top-level HDL wrapper to integrate all modules within the design. Subsequently, the FPGA design is subjected to logic synthesis, followed by place and route operations, where the design is mapped onto FPGA resources, including LUTs, DSPs, and BRAM blocks (17). Finally, a configuration bitstream is generated and programmed into the target FPGA device. During inference, the distilled student CNN processes coronary angiography images and generates a binary classification output, where class 1 denotes plaque-associated angiographic findings and class 0 denotes non-plaque-associated angiographic findings. Table 1 summarises the hardware implementation parameters used for FPGA deployment.

**Table 1:** Hardware implementation parameters used for FPGA deployment.

Parameter	Value
Input image resolution	224 × 224
Gamma correction value	0.8
FPGA clock frequency	100 MHz
Data representation	8-bit-Fixed-point
Target FPGA device	Zynq UltraScale+

## 3 Results

This section presents the performance evaluation of the proposed knowledge-distillation student CNN framework for the classification of plaque-associated angiographic findings from coronary angiography images. The framework is evaluated at both the software and hardware levels. The results are organised into three stages: (i)software-level diagnostic performance evaluation, (ii)Vitis-HLS synthesis analysis, and (iii) hardware evaluation.

### 3.1 Software-level results of the knowledge distilled student CNN

The first stage evaluates the performance of the knowledge distillation technique implemented to train the lightweight distilled student CNN. Using the teacher-student learning paradigm, the student model learns from the teacher model by combining classification and distillation loss functions. This enables the student CNN to achieve high-level diagnostic performance with low computational complexity. The performance of the proposed distilled student CNN is evaluated based on various diagnostic performance metrics. These include accuracy, precision, recall, positive and negative predictive values, and the Matthews correlation coefficient. Both quantitative and qualitative analyses were performed to validate the proposed model.

#### 3.1.1 Effect of knowledge distillation on model performance

The effectiveness of the proposed knowledge-distillation framework was evaluated by comparing the distilled student CNN with baseline architectures, including U-Net, 3D-CNN, and RFDS, using a 90% training and 10% testing split with 10-fold cross-validation. The comparative performance is summarised in Table 2. The segmentation-based teacher network was employed to extract detailed vascular and plaque-related spatial representations from coronary angiography images. These discriminative features were subsequently transferred to the lightweight student CNN through the knowledge distillation process, enabling accurate classification while substantially reducing computational complexity**.** This strategy allows the student model to preserve clinically relevant vascular characteristics without requiring a complex segmentation architecture during inference.

**Table 2:** Comparative diagnostic performance of the proposed distilled student CNN and baseline frameworks using a 90% training split and 10-fold cross-validation.

Methods/metrics	Accuracy (%)	Precision (%)	Recall (%)	NPV	PPV	MCC
Training percentage = 90%						
UNet (8)	96.51	96.40	96.73	0.96	0.96	0.91
3D-CNN (4)	96.33	97.45	94.08	0.94	0.97	0.94
RFDS (5)	93.71	93.83	93.48	0.94	0.94	0.94
Knowledge Distillation (Proposed)	98.64	99.06	97.82	0.98	0.99	0.96
10-fold Cross-Validation						
UNet (8)	95.60 ± 0.42	95.36	96.06	0.93	0.95	0.94
3D-CNN (4)	95.57 ± 0.38	95.67	95.36	0.93	0.96	0.90
RFDS (5)	95.47 ± 0.44	95.54	95.32	0.90	0.96	0.89
Knowledge Distillation (Proposed)	97.53 ± 0.27	97.82	96.94	0.97	0.98	0.95

U-Net and related baseline methods were originally developed for segmentation tasks. They are included in the comparative analysis because the proposed framework employs a segmentation-aware teacher network. Their reported performance is discussed in the context of the corresponding stage of the proposed framework.

The distilled student CNN achieved an **accuracy of 98.64%**, **precision of 99.06%**, **recall of 97.82%**, and an **MCC of 0.96**, demonstrating superior classification performance while maintaining a lightweight architecture suitable for hardware implementation. Compared with the teacher model, which achieved an **accuracy of 97.53%**, **precision of 97.82%**, **recall of 96.94%**, and an **MCC of 0.95**, the proposed knowledge-distillation framework effectively transferred discriminative vascular representations while reducing computational complexity. Consequently, the proposed framework provides high diagnostic performance with reduced computational cost, making it well-suited for lightweight FPGA-oriented and real-time deployment. Since the proposed framework comprises a segmentation-aware teacher network followed by a classification-oriented distilled student CNN, the subsequent comparative analysis evaluates each stage against methods designed for the corresponding task.

#### 3.1.2 Diagnostic performance evaluation

Table 2 compares the performance of the baseline models and the proposed Distilled Student CNN using accuracy, precision, recall, positive predictive value (PPV), negative predictive value (NPV), and Matthews correlation coefficient (MCC). These metrics provide a comprehensive assessment of the classification performance, robustness, and reliability of the models, particularly in the presence of class imbalance. The results demonstrate that the proposed Distilled Student CNN consistently achieved the highest accuracy and MCC among the evaluated models. This indicates its superior capability in classifying plaque-associated angiographic findings from coronary angiography images. To further evaluate the statistical stability and generalisation ability of the proposed framework, 10-fold cross-validation was performed. The results showed low inter-fold variation, indicating stable and reliable performance across validation subsets. The proposed framework achieved an average accuracy of 97.53% with a standard deviation of ±0.27, confirming its robustness and consistency. These findings demonstrate robustness and effectiveness of the proposed framework for classifying plaque-associated angiographic findings. For a fair comparison, all baseline models (UNet, RFDS, and 3D-CNN) were independently implemented and evaluated on the ARCADE Phase-1 Task-2 coronary artery stenosis detection dataset under an identical experimental protocol. The references associated with these baseline models are provided to acknowledge the corresponding methodological approaches adopted during implementation and are not the source of the performance values reported in Table 2. Therefore, all performance metrics presented in Table 2 were obtained from our own experimental implementation under a common evaluation framework.

It should be noted that the proposed framework consists of two complementary stages: a segmentation-aware teacher network and a classification-oriented distilled student CNN. Therefore, segmentation-based baseline methods are included to evaluate the effectiveness of the teacher network, whereas classification-based methods are used to evaluate the final classification performance of the distilled student CNN. Accordingly, the comparative analysis is presented according to the corresponding objective of each stage, thereby providing a technically appropriate and fair, like-for-like comparison.

#### 3.1.3 Qualitative classification results

**Figure 5:**
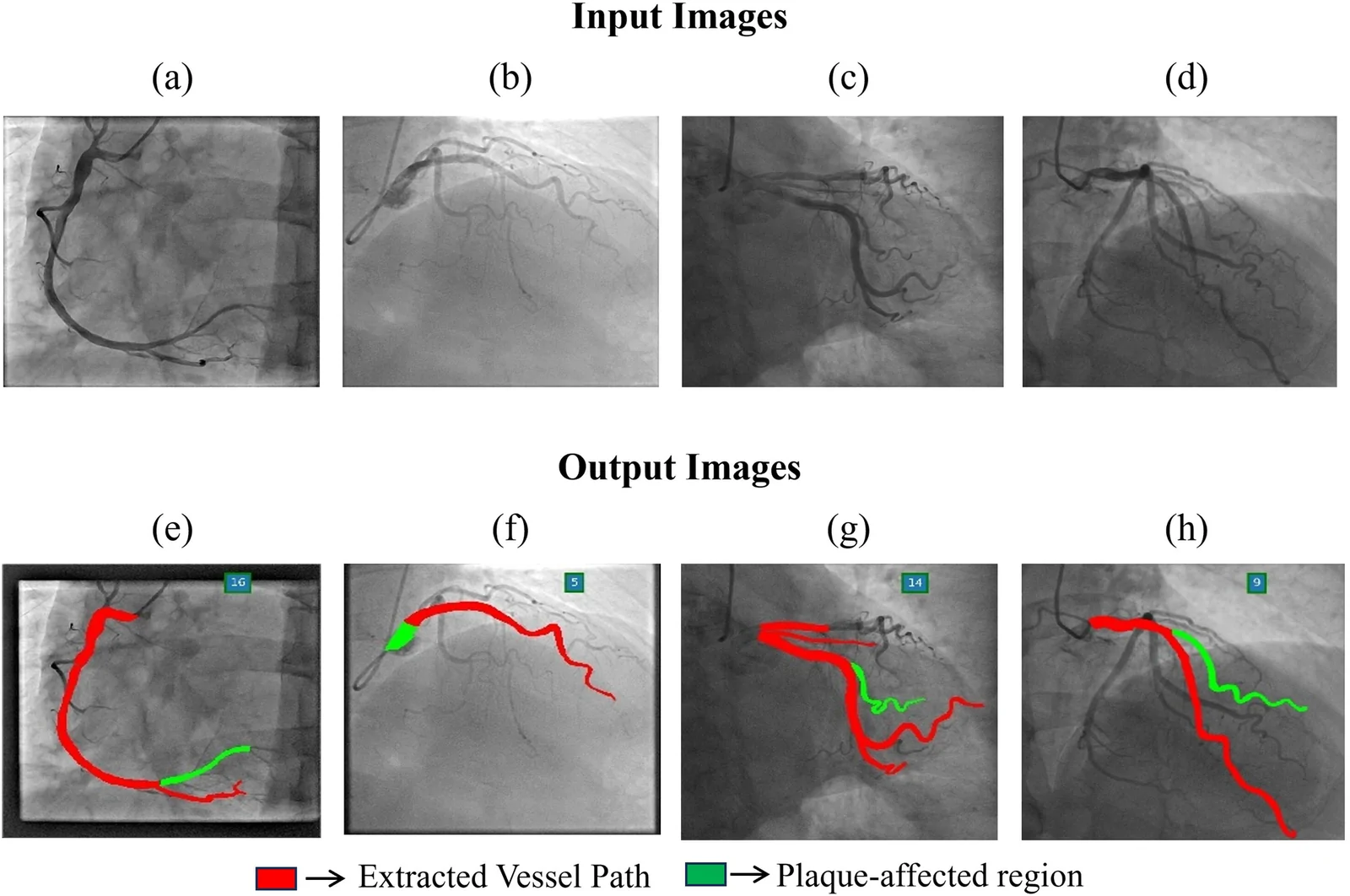
Qualitative visualisation of the proposed knowledge distillation framework showing representative coronary angiography input images **(a)–(d)** and the corresponding model output images **(e)–(h).**

Qualitative results are presented to demonstrate the effectiveness and practical applicability of the proposed Distilled Student CNN. Figure 5 illustrates representative coronary angiography images used for qualitative evaluation. Panels (a–d) show the original input images from the ARCADE dataset, whereas panels (e–h) present the corresponding outputs generated by the proposed framework. The visual results demonstrate that the distilled student CNN effectively highlights coronary vessel trajectories (shown in red) and angiographic regions associated with atherosclerotic findings (shown in green), supporting accurate classification while preserving the anatomical structure of the coronary arteries. These qualitative observations are consistent with the quantitative results presented in Table 2 and demonstrate the capability of the proposed framework to identify diagnostically relevant vascular patterns for coronary atherosclerotic classification.

#### 3.1.4 Ablation studies for the proposed model

To comprehensively evaluate the contribution of each component in the proposed framework, the ablation study was expanded in Table 3. To systematically investigate the effects of preprocessing, knowledge distillation, feature alignment, distillation temperature, magnitude-based pruning, and signed INT8 post-training quantization. Each experiment was designed by progressively incorporating a single component while maintaining all remaining training settings unchanged, thereby isolating its individual contribution to the overall classification performance.

**Table 3:** Comprehensive ablation study evaluating the individual and cumulative contributions of the proposed framework.

Configuration/stage	Loss terms included	Temperature (T)	Accuracy (%)	Precision (%)	Recall (%)	MCC
Baseline Student CNN	L_CE_	N/A	93.71	93.83	93.48	0.89
+ Gamma Correction	L_CE_	N/A	94.50	94.72	94.10	0.91
+ GAN Augmentation	L_CE_	N/A	94.80	95.02	94.36	0.91
+ Standard KD (Without Feature Alignment)	L_CE_ + L_KL_	T = 1	95.62	95.90	95.12	0.92
+ Softened KD	L_CE_ + L_KL_	T = 4	97.41	97.85	96.92	0.94
+ Feature Alignment Loss	L_CE_ + L_KL_ + L_FA_	T = 4	98.85	99.12	98.10	0.97
+ 30% Magnitude-Based Pruning	L_CE_ + L_KL_ + L_FA_	T = 4	98.71	99.10	97.91	0.96
Full Framework (Pruned + Signed INT8 PTQ)	L_CE_ + L_KL_ + L_FA_	T = 4	98.64	99.06	97.82	0.96

N/A indicates that temperature scaling is not applicable before knowledge distillation because the temperature parameter is used only in the teacher–student KL divergence loss formulation.

L_CE_, cross-entropy loss; L_KL_, Kullback–Leibler divergence loss; L_FA_, feature alignment loss; T, distillation temperature; KD, knowledge distillation; PTQ, post-training quantization; MCC, Matthews correlation coefficient.

#### 3.1.5 Progressive performance comparison of the teacher and student models

**To further evaluate the effectiveness of the proposed optimization strategy, a progressive performance comparison was conducted using the teacher network, baseline student, knowledge-distilled student, 30% magnitude-pruned student, and the final signed INT8 post-training quantized (PTQ) student model.** The results demonstrate that the **knowledge-distilled student** achieved the highest floating-point classification accuracy of **98.85%**, while the **pruned** and **quantized** models retained **98.71%** and **98.64%** classification accuracy, respectively, with substantial reductions in model size and memory footprint. As shown in Table 4, knowledge distillation substantially improved the performance of the lightweight student model. Although magnitude-based pruning and signed INT8 post-training quantization introduced only marginal reductions in classification accuracy, they significantly reduced the model storage requirements while preserving the overall predictive performance, thereby enabling efficient FPGA deployment.

**Table 4:** Progressive comparison of the teacher network, baseline student, knowledge-distilled student, pruned student, and quantized student models.

Model stage	Numerical precision	Model representation/storage	Classification accuracy (%)	Accuracy variation vs. peak knowledge-distilled model (%)
Teacher Network	32-bit Floating-Point (FP32)	Software Training (NVIDIA RTX 5000 Ada GPU with 32 GB Memory)	99.12	+0.27 (Teacher Reference)
Baseline Student (Without Knowledge Distillation)	32-bit Floating-Point (FP32)	193.38 KB (Target FPGA Deployment)	93.71	−5.14
Knowledge-Distilled Student	32-bit Floating-Point (FP32)	193.38 KB (Target FPGA Deployment)	98.85	Base (0.00)
30% Magnitude-Pruned Student	32-bit Floating-Point (FP32)	138.62 KB (135.37 KiB) (Target FPGA Deployment)	98.71	−0.14
Final Quantized Student	Signed 8-bit Integer (INT8)	34.65 KB (33.84 KiB) (Xilinx Zynq UltraScale + FPGA)	98.64	−0.21

The baseline student model contained **49,505 parameters** stored using **32-bit Floating-Point (FP32)** precision. Magnitude-based pruning reduced the parameter count to **34,654** (approximately **30% reduction**). Subsequently, **Signed INT8 Post-Training Quantization (PTQ)** using **MinMax calibration** further reduced the model storage footprint to **34.65 KB (33.84 KiB)**, corresponding to an **82.08% overall memory reduction** compared with the original FP32 baseline. The INT8 quantization stage alone reduced the memory requirement of the pruned FP32 model by **75%**. The final optimized model achieved **98.64% classification accuracy**, with an **inference latency of 10.6 µs** and a **throughput of 94,339 inferences/s** on the **Xilinx Zynq UltraScale** **+** **FPGA**.

#### 3.1.6 Confusion matrix analysis

**Figure 6:**
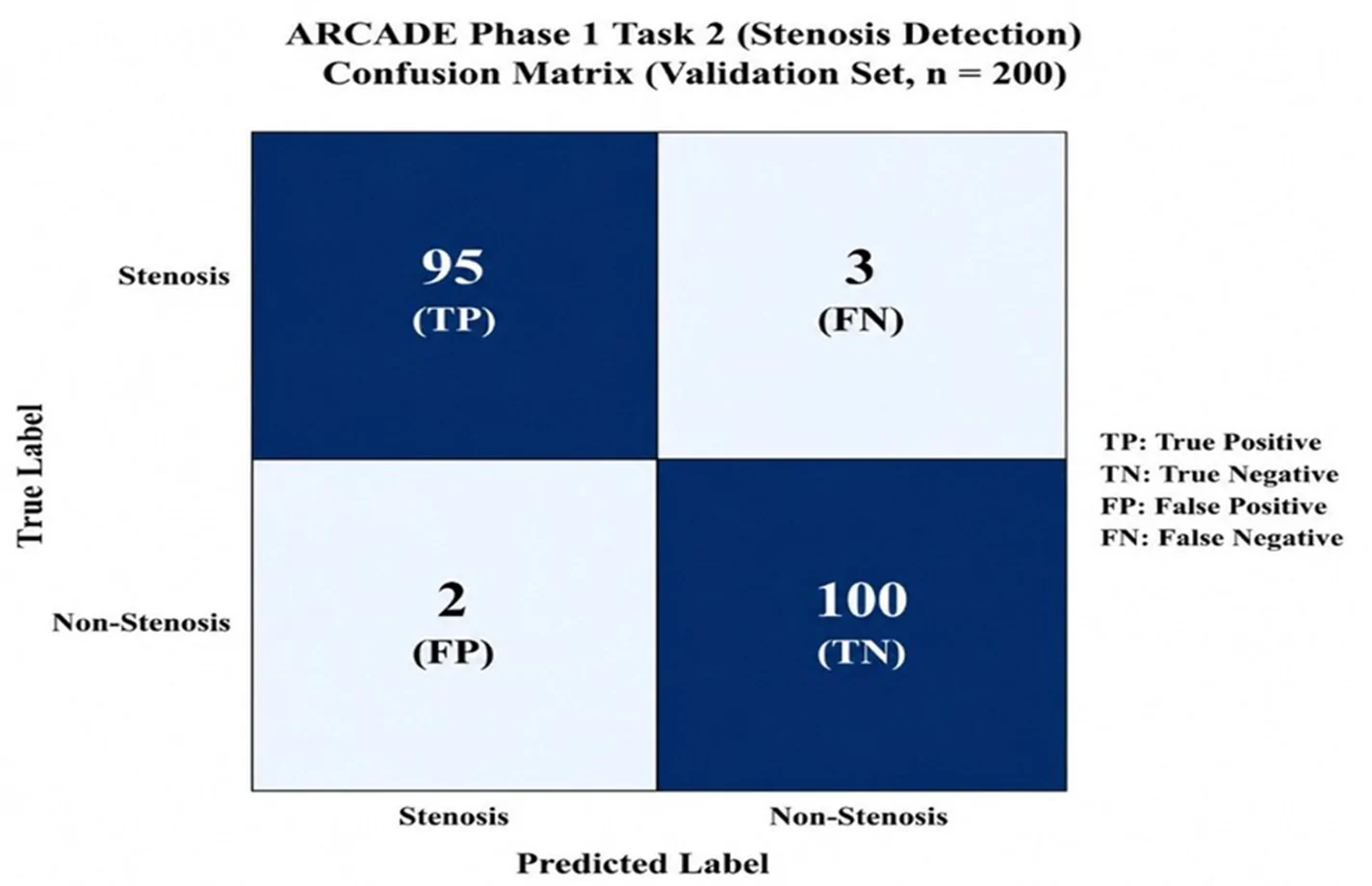
Confusion matrix of the proposed knowledge-distilled student CNN evaluated on the ARCADE phase 1 task 2 validation set (*n* = 200).

The confusion matrix of the proposed knowledge-distilled student CNN on the ARCADE Phase 1 Task 2 validation set (*n* = 200) is presented in Figure 6. The model correctly classified 95 stenosis (true positives) TP and 100 non-stenosis cases (true negatives) TN, with only 3 FN false negatives and 2 FP false positives. The low number of misclassifications indicates balanced classification performance across both classes and supports the reliability of the proposed framework for automated classification of plaque-associated angiographic findings. However, these prediction errors also have important clinical implications. While false-negative predictions may delay disease detection by failing to identify stenotic cases, false-positive predictions may lead to unnecessary follow-up investigations. These findings highlight the importance of using the proposed framework as a clinical decision-support tool to assist clinicians rather than as a replacement for clinical judgment.

#### 3.1.7 Receiver operating characteristic (ROC) analysis of the proposed framework

**Figure 7:**
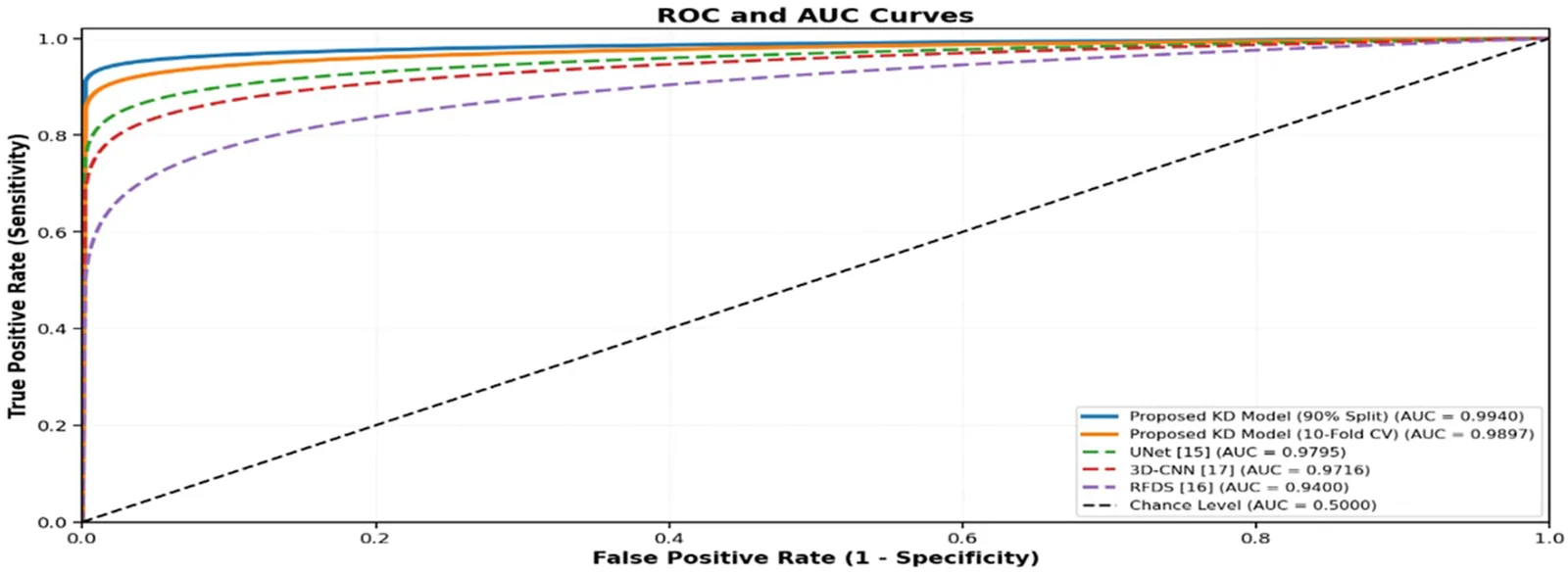
Receiver operating characteristic (ROC) curves comparing the proposed knowledge distillation model evaluated using the 90% training and 10% testing split and 10-fold cross-validation with the baseline models (UNet, 3D-CNN, and RFDS). The proposed model achieved the highest discriminative performance with an AUC of 0.9940 using 90% training and 10% testing split and a mean area under the receiver operating characteristic curve (AUC) of 0.9897 under 10-fold cross-validation.

Receiver Operating Characteristic (ROC) analysis was performed to further evaluate the discriminative capability of the proposed framework. The corresponding Area Under the Receiver Operating Characteristic Curve (AUC). As illustrated in Figure 7, the proposed Knowledge Distillation model achieved an Area Under the Receiver Operating Characteristic Curve (AUC) of 0.9940 using the 90% training and 10% testing split and a mean AUC of 0.9897 under 10-fold cross-validation, outperforming the baseline models (UNet, RFDS, and 3D-CNN) in terms of discriminative performance. These results demonstrate the excellent discriminative capability and robustness of the proposed framework across different evaluation protocols.

### 3.2 Hardware synthesis results using Vitis HLS

After validating the software-level diagnostic performance of the proposed Knowledge-distillation student CNN, the trained inference model was translated into a hardware implementation using Vitis HLS. The section evaluates the hardware-oriented characteristics of the distilled model prior to FPGA deployment, including functional correctness, execution latency, resource utilisation and synthesis efficiency. These results demonstrate that the proposed lightweight architecture preserves its classification capability while satisfying the requirements for efficient real-time FPGA implementation.

#### 3.2.1 RTL co-simulation verification

**Figure 8:**
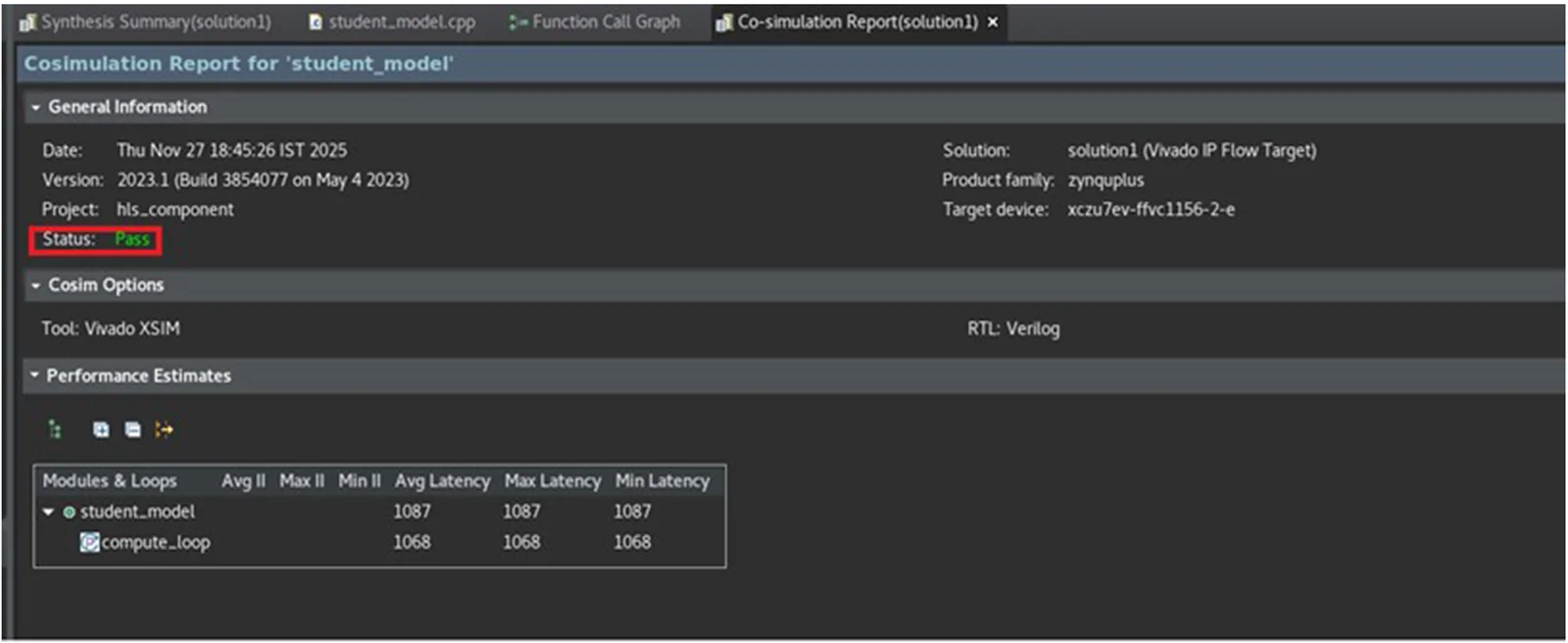
Vivado XSIM RTL co-simulation report showing successful functional verification and latency estimation of the proposed knowledge-distilled student CNN accelerator.

RTL co-simulation was performed using Vivado XSIM to verify the functional correctness between the HLS-generated and RTL implementation and the corresponding C++ reference model. The simulation completed successfully with **PASS** status, confirming that the synthesized RTL produces outputs consistent with the software implementation. The Co-simulation report further provides cycle-accurate timing information, with an overall inference latency of 1,087 clock cycles, including approximately 1,068 cycles for the main computational loop. These results confirm deterministic hardware execution and validate the correctness of the generated RTL before FPGA synthesis and implementation, as illustrated in Figure 8.

Following successful RTL verification, the design proceeded through the Vitis HLS synthesis flow to generate the final synthesized hardware implementation. Consequently, the latency values reported in Figure 8 correspond to the RTL co-simulation results obtained during the HLS synthesis flow and were used exclusively to verify the functional correctness of the generated RTL design. These RTL co-simulation results are intended solely for functional verification and should not be interpreted as the final hardware performance results reported for the implemented FPGA accelerator.

#### 3.2.2 RTL waveform validation

**Figure 9:**
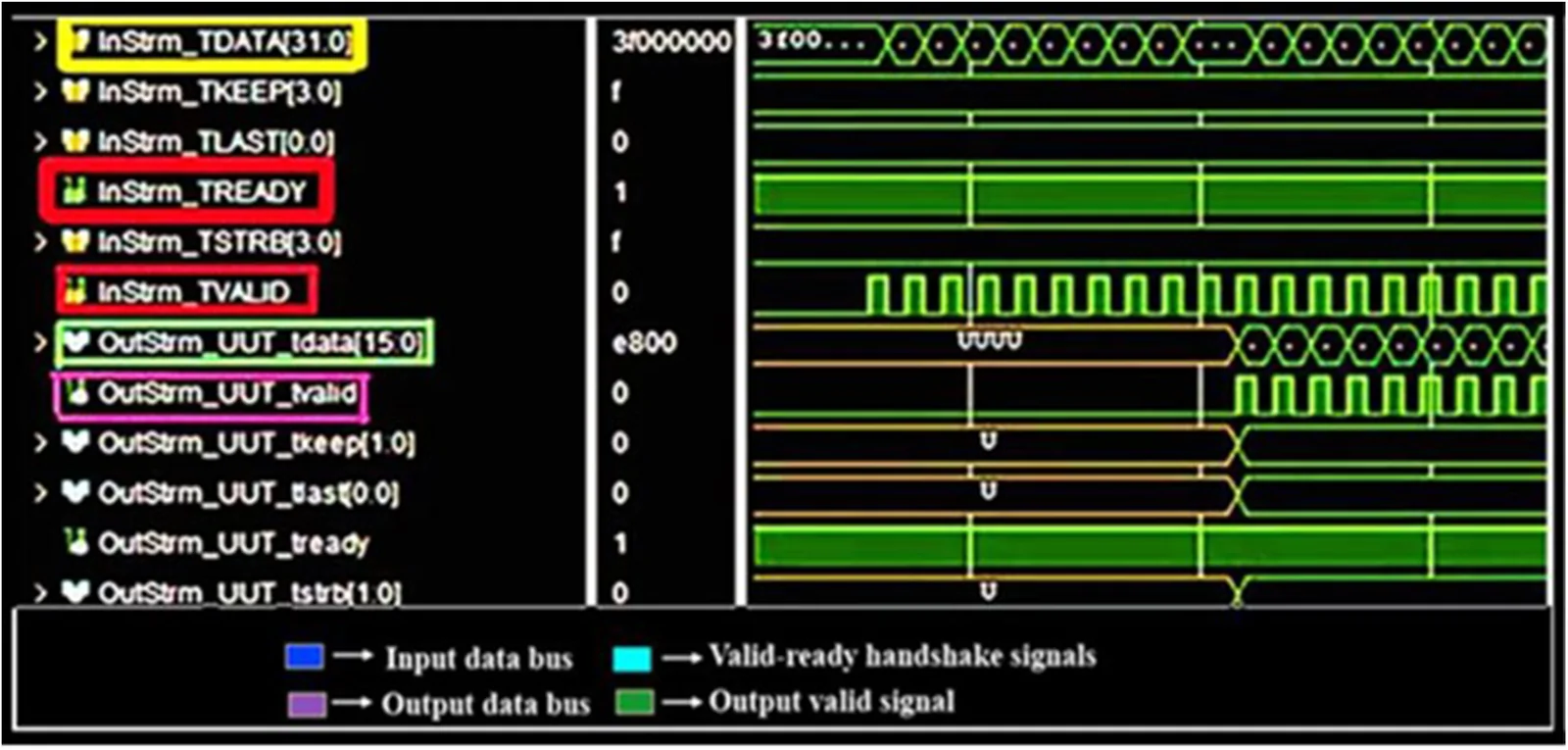
RTL co-simulation waveforms obtained from Vivado XSIM showing successful AXI-stream communication and functional validation of the synthesized knowledge-distilled student CNN accelerator.

To further validate the synthesized hardware implementation, RTL waveform analysis was performed using Vivado XSIM. Figure 9 presents the timing behaviour of the AXI-Stream interface during inference. The waveform demonstrates correct synchronization between the TVALID, TREADY, and TDATA signals, demonstrating successful data transfer without timing violations, communication stalls, or protocol errors. The stable valid-ready handshake verifies that input data are transferred reliably through the accelerator pipeline and processed in the expected sequence. These observations confirm the functional correctness and timing stability of the synthesized RTL implementation, further demonstrating the suitability of the proposed knowledge-distilled student CNN for reliable FPGA deployment.

As illustrated in Figure 9, the AXI-Stream interface maintains correct synchronization throughout the inference process. The coordinated assertion of TVALID and TREADY enables uninterrupted data transmission, while the stable TDATA waveform confirms accurate propagation of input data through the hardware pipeline. No communication stalls or timing inconsistencies are observed during simulation, indicating deterministic execution of the synthesized accelerator. These results further confirm the correctness and reliability of the proposed FPGA implementation.

#### 3.2.3 Module-level latency and resource distribution

To analyse the computational behaviour of the synthesized accelerator, the module-level latency and hardware resource distribution obtained from Vitis HLS are presented in Table 5. The top-level **student_model** module requires **1,060 clock cycles** to complete one inference, while the **compute_loop**, which performs the core convolution and feature extraction operations, accounts for **1,045 clock cycles** of the total execution time. This indicates that the majority of the inference latency is attributed to the core CNN computation, whereas the remaining latency is associated with top-level control and interface management. The synthesized top-level accelerator utilizes **809 BRAMs** and **1,280 DSP slices**, with **no URAM resources required**, demonstrating efficient utilization of on-chip memory and computational resources for the proposed lightweight knowledge-distilled CNN.

**Table 5:** Module-level latency and hardware resource utilisation of the synthesized knowledge-distilled student CNN obtained from Vitis HLS.

Module	Latency (cycles)	BRAM (total)	DSP (total)	URAM	Role
Student_model	1,060	809	1,280	0	Top-level control wrapper
Compute_loop	1,045	–	–	0	Core CNN computation

The **compute_loop** represents the primary computation kernel responsible for convolution and feature extraction and contributes **1,045 clock cycles** to the overall latency. Hardware resources (BRAM, DSP, and URAM) are reported at the **student_model** level because Vitis HLS provides resource utilisation for the synthesized top-level accelerator rather than for individual internal functions.

#### 3.2.4 End-to-end latency and throughput analysis based on Vitis HLS estimates

The end-to-end inference latency and throughput of the proposed FPGA accelerator were estimated using Vitis HLS synthesis results. Based on the final synthesized hardware implementation generated by Vitis HLS, the Knowledge-distilled student CNN model requires 1,060 clock cycles to process a single coronary angiography image at an operating frequency of 100 MHz. The corresponding latency and throughput are calculated in Equation 14.

$$Latency=\frac{1060}{100\times 10^{6}}=10.6\;\mu s\;\mathrm{per}\;\mathrm{image}$$ (14)

This corresponds to an end-to-end inference latency of approximately 10.6 *μ*s per image. The throughput of the accelerator is estimated using Equation 15.

$$Throughput=\frac{100\times 10^{6}}{1060}=94339\;\mathrm{images}\;\mathrm{per}\;\mathrm{second}$$ (15)

The estimated throughput is approximately 94,339 images per second. These results demonstrate that the synthesized knowledge-distilled student CNN accelerator achieves low inference latency together with high throughput, making it suitable for real-time FPGA deployment for coronary angiography image classification.

The reported 10.6 µs represents the kernel-level inference latency of the synthesized FPGA accelerator and excludes host-to-FPGA communication, AXI Direct Memory Access (AXI-DMA), external memory transfers, preprocessing, output communication, and other system-level input/output overheads. Since end-to-end board-level latency measurements were not performed in this study, the reported latency should not be interpreted as the overall system latency. Similarly, the reported throughput of 94,339 inferences/s represents the Peak On-Chip Kernel Throughput derived from the synthesized kernel execution cycle count and operating clock frequency, rather than an experimentally measured end-to-end system throughput.

Following Vitis HLS synthesis and performance evaluation, the generated RTL design is exported to the Vivado Design Suite for FPGA implementation. The subsequent section presents the post-implementation hardware results, including resource utilization, timing analysis, and device-level validation on the target Zynq UltraScale + FPGA platform.

### 3.3 Vivado FPGA results: hardware implementation

The synthesized RTL design generated by Vitis HLS was implemented on the target Zynq UltraScale + MPSoC platform using the Vivado Design Suite. This section presents the post-implementation hardware results, including FPGA resource utilization, timing performance, and implementation characteristics of the proposed knowledge-distilled student CNN accelerator.

#### 3.3.1 FPGA resource utilisation

**Figure 10:**
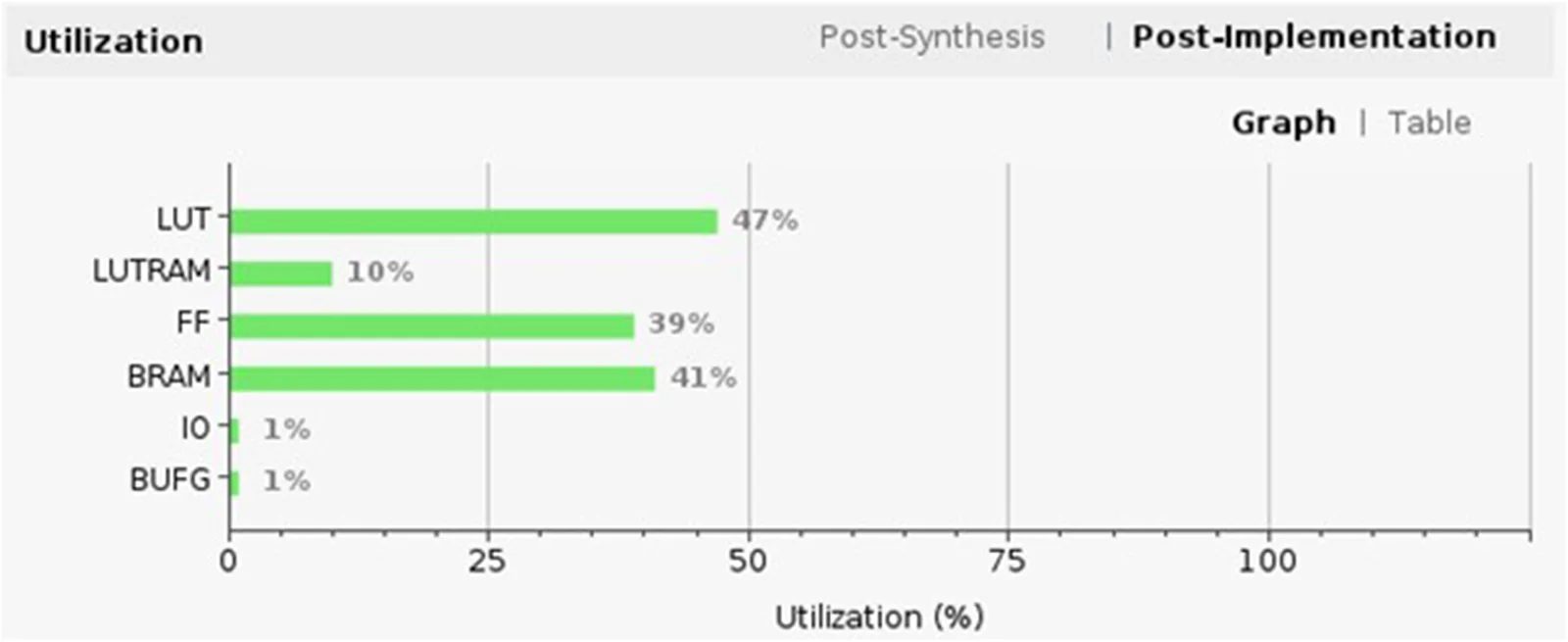
Post-implementation FPGA resource utilisation of the proposed knowledge-distilled student CNN accelerator on the Zynq UltraScale + MPSoC platform.

The post-implementation FPGA resource utilisation of the proposed knowledge-distilled student CNN accelerator on the target Zynq UltraScale + MPSoC platform is presented in Figure 10. The implementation results demonstrate efficient utilisation of the available FPGA resources. BRAM resources are predominantly allocated for storing intermediate feature maps, whereas LUTs, LUTRAM, Flip-Flops (FFs), and global clock buffers (BUFGs) are used for implementing the computational logic, pipeline registers, control circuitry, and clock distribution. The relatively low utilisation of LUTRAM and BUFG resources indicates minimal control and clock-management overhead, while the higher BRAM utilisation reflects the memory requirements of the CNN inference pipeline.

Table 6 summarises the utilisation percentages of the major FPGA resources together with their functional roles. The implementation utilises **LUTs (47%)**, **Flip-Flops (39%)**, and **BRAMs (41%)**, all of which remain within the available device capacity, demonstrating an efficient balance between computational logic and memory resources. In contrast, **LUTRAM (10%)** and **BUFG (1%)** exhibit comparatively low utilisation, confirming that only a small proportion of resources is required for control and clock-management functions. Overall, these results demonstrate that the proposed knowledge-distilled student CNN accelerator achieves an efficient hardware implementation with balanced resource allocation, making it suitable for deployment on resource-constrained FPGA platforms. To avoid ambiguity, the hardware resource utilization reported in this study corresponds exclusively to the final optimized knowledge-distilled, 30% magnitude-pruned, and signed INT8 post-training quantized accelerator obtained from the Vivado post-implementation report. The previously generated intermediate Vitis HLS synthesis estimates were used only during design-space exploration and are not representative of the final implemented hardware. Therefore, only the validated post-implementation resource utilization is presented to accurately reflect the implemented FPGA accelerator.

**Table 6:** Post-implementation FPGA resource utilisation and availability of the proposed knowledge-distilled student CNN accelerator on the target Zynq UltraScale + MPSoC platform.

Resource	Used	Available	Utilisation (%)
LUT	107,874	230,400	46.82
LUTRAM	10,424	101,760	10.24
FF	178,143	460,800	38.66
BRAM	128	312	41.03
IO	2	360	0.56
BUFG	2	544	0.37

The hardware resource utilization reported in this study corresponds exclusively to the final optimized knowledge-distilled, 30% magnitude-pruned, and signed INT8 post-training quantized accelerator obtained from the Vivado post-implementation report. The previously generated intermediate Vitis HLS synthesis estimates were used only during design-space exploration and are not representative of the final implemented hardware. Therefore, only the validated post-implementation resource utilization is presented to accurately reflect the implemented FPGA accelerator.

#### 3.3.2 Detailed FPGA resource statistics

To provide detailed post-implementation device-level resource statistics, Table 6 summarizes the used, available, and utilisation percentages of the major FPGA resources obtained after implementation whereas Table 7 shows the interpretation of resource utilization. All major resources utilise less than 50% of the available device capacity, indicating that the proposed knowledge-distilled student CNN accelerator fits comfortably within the target Zynq UltraScale + MPSoC while leaving sufficient hardware margin for future system expansion.

**Table 7:** Interpretation of the post-implementation FPGA resource utilisation of the proposed knowledge-distilled student CNN accelerator.

Resource	Utilisation (%)	Interpretation
LUT	∼47%	Logic utilisation remains below half of the available resources
Flip-Flops	∼39%	Moderate register usage indicates a balanced pipeline design
BRAM	∼41%	Efficient on-chip memory utilisation for feature storage
LUTRAM	∼10%	Limited distributed memory usage
BUFG	∼1%	Minimal clock buffer consumption
IO	∼1%	Low I/O utilisation

#### 3.3.3 Clock performance analysis

Table 8 summarises the clock performance of the implemented design. The programmable logic (PL) system clock (clk_pl_0) operates at 100 MHz with a clock period of 10 ns, whereas the HLS kernel execution clock (ap_clk_0) operates with a clock period of 7 ns, corresponding to a maximum operating frequency of 142.86 MHz. The achieved 7 ns clock period satisfies the design timing constraints and provides a positive timing margin of approximately 2.7 ns, confirming successful timing closure and stable operation of the proposed knowledge-distilled student CNN accelerator.

**Table 8:** Clock performance summary of the implemented knowledge-distilled student CNN accelerator.

Clock domain	Period (ns)	Frequency (MHz)	Role
ap_clk_0	7	142.86	HLS kernel execution clock
clk_pl_0	10	100	Programmable logic (PL) System clock

#### 3.3.4 Power consumption analysis

Table 9 presents the post-implementation on-chip power consumption of the proposed Knowledge-distilled student CNN accelerator on the target Zynq UltraScale + MPSoC platform. The total on-chip power consumption is **4.41 W**, comprising **3.712 W (84.17%)** dynamic power and **0.699 W (15.85%)** static power. The dominance of dynamic power indicates that most of the energy is consumed during active switching and computation, while the relatively lower static power demonstrates limited leakage in the implemented design.

**Table 9:** Post-implementation on-chip power consumption of the proposed knowledge-distilled student CNN accelerator on the target Zynq UltraScale + MPSoC platform.

Power component	Power consumption (W)	Power distribution (%)	Description
Total On-Chip Power	4.41	100.00%	Overall power consumption
Dynamic Power	3.712	84.17%	Power due to switching activity
Static Power	0.699	15.85%	Leakage and static components
Processing System (PS)	2.639	59.84%	ARM cores and PS infrastructure
Programmable Logic (PL)	0.599	13.58%	FPGA fabric contribution
Clocks	0.394	8.93%	Clock distribution network
Signals	0.264	5.99%	Signal toggling
Logic	0.325	7.37%	Combinational and sequential logic
BRAM	0.089	2.02%	On-chip memory
I/O	< 0.001	< 0.03%	External interfaces

The power distribution further shows that the **Processing System (PS)** consumes **2.639 W (59.84%)**, whereas the **Programmable Logic (PL)** consumes only **0.599 W (13.58%)**, indicating that the proposed Knowledge-distilled student CNN accelerator occupies only a modest proportion of the programmable logic power. In addition, the clock network contributes **0.394 W (8.93%)**, while signal routing accounts for **0.264 W (5.99%)** of the total on-chip power. These results demonstrate that the proposed knowledge-distilled student CNN accelerator achieves efficient power utilization while maintaining real-time inference capability on the target Zynq UltraScale + MPSoC platform.

### 3.4 Timing closure and end-to-end latency analysis

Table 10 shows the timing characteristics and execution latency of the synthesised student model. The design has achieved a positive timing slack of +2.7 ns, which indicates the target clock constraint has been met. The accelerator has achieved a total inference latency of 1,060 clock cycles. This corresponds to 10.6 μs latency at a clock frequency of 100 MHz. The compute loop is fully pipelined with an initiation interval of 1. This shows the effectiveness of the proposed architecture in achieving low latency in real-time inference on FPGAs. The hardware efficiency of the proposed distilled student CNN accelerator is evaluated to measure how effectively FPGA logic resources are utilized to achieve high inference throughput. Hardware efficiency is defined as the ratio between the achieved throughput and the number of LUTs used in the FPGA implementation. Using this measured throughput of 94,339 and LUT utilisation of 343,935, the hardware efficiency of the proposed accelerator is evaluated using Equation 16.

$$Hardware\;Efficiency=\frac{Throughput}{LUTs}=\frac{94339}{343935}=0.274\;images/s/LUT$$ (16)

**Table 10:** Timing closure and end-to-end latency characteristics of the proposed knowledge-distilled student CNN accelerator.

Parameter	Value	Explanation
Target FPGA device	xczu7ev-ffvc1156-2-e	Zynq UltraScale + platform
Target clock period	10.00 ns	Design constraint (100 MHz)
Achieved clock period	7.30 ns	Achieved after place-and-route
Timing margin (slack)	+2.70 ns	Positive margin → timing requirements met
Total latency (cycles)	1,060 cycles	End-to-end inference latency
Total latency (time)	10.6 μs	At 100 MHz clock frequency
Initiation interval (top)	1,061	Non-pipelined top module
Core loop pipelining	Enabled	compute_loop pipelined

Unlike the Vitis HLS estimates presented in Section 3.2, the timing values reported in Table 9 are obtained from the post-implementation Vivado timing analysis after synthesis, placement, and routing. Therefore, minor differences in latency and compute loop cycles are expected due to implementation-level optimizations.

These results indicate that the proposed Knowledge-distilled student CNN accelerator achieves successful timing closure and low inference latency while maintaining efficient FPGA resource utilisation and high throughput suitable for real-time biomedical applications.

To further evaluate the performance of the proposed Knowledge-distilled student CNN accelerator, its latency and throughput are compared with those of recent FPGA-based deep learning accelerators reported in the literature, as presented in Table 11. The reported Kernel-Level Inference Latency represents the execution time of the FPGA accelerator after model initialization. It excludes image transfer from external memory, preprocessing, host-to-FPGA communication, and output transfer. Therefore, the reported latency should be interpreted as kernel-level hardware performance rather than end-to-end system latency*.*

**Table 11:** Qualitative comparison of FPGA inference performance with existing hardware accelerators.

Reference	Application/method	FPGA platform	Latency	Throughput	Power/power efficiency
Irmak and Alachiotis et al. (20)	LeNet CNN Accelerator	Xilinx Artix-7 XC7A100 T (Nexys4 DDR)	70.2 µs	14,000 images/s	628 mW
Kim and Choi et al. (12)	Reconfigurable ResNet-20 CNN Accelerator	Xilinx PYNQ-Z1	–	9.17 FPS	43.9 GOPS/W
Al Amin et al. (13)	HePiLUT CNN Accelerator	Xilinx ZCU102	7.716 µs	129,600 FPS	3.45 W
Hong et al. (18)	Mobile-X (MobileNet-V1)	Xilinx XCZU9EG (ZCU102)	–	190.9 FPS (108.3 GOPS)	18.20 GOPS/W
AbdElkarim et al. (14)	ResNet-18 + Vitis AI	Xilinx ZCU104	5.89 ms	169.65 FPS	15.54 W
Cratere et al. (24)	Patch-Net (Vitis AI + DPU)	Ultra96-V2 (Zynq UltraScale + MPSoC)	17.5 ms	57.14 FPS	2.5 W
Proposed Work	Knowledge-Distilled Hardware-Aware CNN	XCZU7EV Zynq UltraScale + MPSoC	10.6 µs	94,339 images/s	4.41 W

Throughput and power metrics are reported exactly as presented in the respective publications (FPS (Frames Per Second), images/s (Images Per Second), GOPS (Giga Operations Per Second), mW (Milliwatts)). Therefore, direct numerical comparisons should be interpreted with caution because different FPGA platforms, CNN architectures, datasets, and evaluation methodologies.

To provide a fair interpretation of the comparative hardware results, it should be noted that direct comparisons among FPGA implementations should be interpreted qualitatively because the reported results are influenced by differences in datasets, input resolutions, network architectures, numerical precision, FPGA platforms, operating frequencies, and evaluation methodologies. Therefore, the comparison presented in Table 11 is intended to provide a qualitative assessment of the proposed FPGA accelerator relative to previously reported FPGA implementations rather than a strict quantitative benchmark.

The comparison demonstrates that the proposed knowledge-distilled student CNN accelerator achieves lower inference latency and higher throughput than several existing FPGA-based deep learning accelerators. The achieved inference latency of 10.6 μs and throughput of 94,339 images/s demonstrate that the distilled student architecture enables efficient real-time FPGA inference for the classification of plaque-associated angiographic findings in coronary angiography.

#### 3.4.1 RTL functional verification

**Figure 11:**
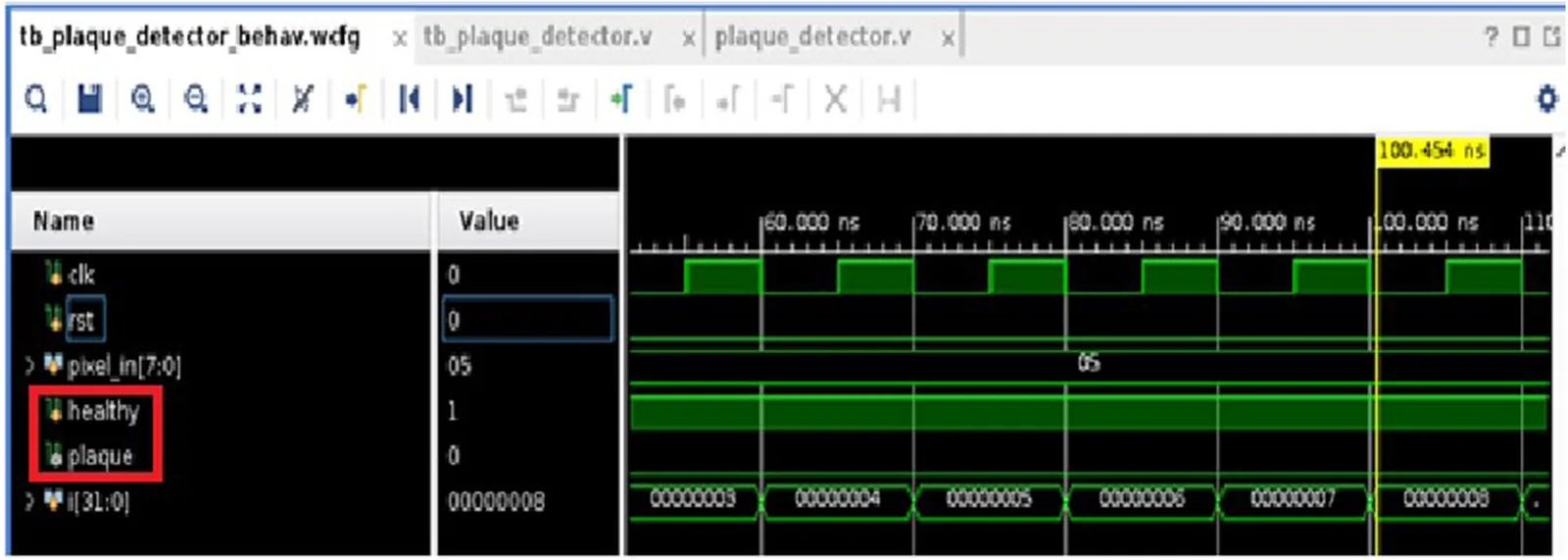
RTL waveform obtained from post-synthesis simulation verifying binary classification outputs of the proposed knowledge-distilled student CNN accelerator, where healthy = ‘1’, plaque = ‘0’.

The correctness of the generated hardware design is validated through RTL waveform simulation, as shown in Figure 11. The RTL waveform confirms the correct sequential processing of input data and the generation of classification outputs. The Healthy and Plaque output signals follow a mutually exclusive binary decision scheme, ensuring deterministic and unambiguous classification results. Furthermore, the absence of glitches, metastability, or unstable transitions confirms correct synchronous operation and the functional correctness of the implemented FPGA accelerator.

## 4 Discussion

The proposed knowledge-distillation-based hardware-aware CNN demonstrates that accurate **classification of plaque-associated angiographic findings** can be achieved while enabling efficient FPGA deployment for real-time coronary angiography analysis. By integrating segmentation-aware teacher learning, knowledge distillation, model pruning, signed INT8 quantization, and FPGA-oriented optimization, the proposed framework achieves a favourable balance between classification performance, computational efficiency, hardware resource utilization, low latency, and power consumption, making it suitable for embedded medical applications.

The obtained results demonstrate the feasibility of integrating knowledge distillation with FPGA-oriented optimization for coronary angiography analysis. However, the findings should be interpreted in the context of the study design. The proposed framework was evaluated using the publicly available **ARCADE Phase-1 Task-2 Coronary Artery Stenosis Detection** dataset. Since the primary objective of this study was to develop and validate a hardware–software co-design framework for accurate coronary angiography classification and efficient FPGA implementation, external validation using independent multi-centre datasets acquired from different institutions, imaging devices, and patient populations was beyond the scope of the present study. Furthermore, because coronary angiography primarily visualizes the vessel lumen rather than the arterial wall, the predicted positive class should be interpreted as **plaque-associated angiographic findings** rather than direct visualization of atherosclerotic plaque morphology.

The publicly available ARCADE Phase-1 Task-2 dataset does not provide patient-level identifiers or detailed lesion-level annotations. Consequently, patient-wise evaluation and several clinically relevant analyses, including patient-level confidence interval estimation, probability calibration, lesion-level sensitivity assessment, and comprehensive false-negative analysis, could not be performed within the scope of this study. Nevertheless, image-level data partitioning was performed before GAN augmentation, and all GAN-generated samples were created exclusively from the training partition, while the validation and test datasets contained only original ARCADE images, thereby minimizing the possibility of data leakage during model evaluation. Future work will focus on external multi-centre clinical validation using independent datasets acquired from different institutions and imaging devices, evaluation using datasets with patient-level identifiers, comparison with clinician interpretation, integration with complementary imaging modalities such as intravascular ultrasound (IVUS), optical coherence tomography (OCT), and coronary computed tomography angiography (CCTA), as well as further optimization for low-power edge deployment and multi-class coronary artery disease assessment.

## 5 Conclusion

This work presented a **knowledge-distillation-based hardware-aware CNN framework** for the classification of **plaque-associated angiographic findings** in coronary angiography images and its efficient deployment on a **Zynq UltraScale** **+** **MPSoC FPGA**. By combining segmentation-aware teacher–student learning with FPGA-oriented implementation, the proposed framework achieved high classification performance while maintaining a compact and hardware-efficient architecture. Experimental evaluation demonstrated a **classification accuracy of 98.64% and an AUC of 0.9940** using a 90% training and 10% testing split configuration and an average **97.53%** **±** **0.27% accuracy** and a mean AUC of 0.9897 under 10-fold cross-validation, confirming robust and reliable performance. The post-implementation FPGA design achieved successful timing closure with an achieved clock period of **7.30 ns** and **+2.70 ns** timing slack while requiring only **46.82% LUT**, **38.66% FF**, and **41.03% BRAM** resources without URAM utilisation. Furthermore, the accelerator achieved a kernel-level inference latency of **10.6 μs (1,060 clock cycles)**, a peak On-Chip Kernel Throughput of **94,339** inferences/s, and a total on-chip power consumption of **4.41 W**, demonstrating its suitability for real-time FPGA deployment. Overall, the proposed framework effectively balances diagnostic accuracy with hardware efficiency, making it a practical solution for real-time computer-aided analysis of coronary angiography images in resource-constrained clinical environments.

## Data Availability

The original contributions presented in the study are included in the article/Supplementary Material, further inquiries can be directed to the corresponding author.
